# Carbon-Based Nanocatalysts (CnCs) for Biomass Valorization and Hazardous Organics Remediation

**DOI:** 10.3390/nano12101679

**Published:** 2022-05-14

**Authors:** Dimitrios A. Giannakoudakis, Foteini F. Zormpa, Antigoni G. Margellou, Abdul Qayyum, Ramón Fernando Colmenares-Quintero, Christophe Len, Juan Carlos Colmenares, Konstantinos S. Triantafyllidis

**Affiliations:** 1 Department of Chemistry, Aristotle University of Thessaloniki, 54124 Thessaloniki, Greece; dagchem@gmail.com (D.A.G.); foteinizf@chem.auth.gr (F.F.Z.); amargel@chem.auth.gr (A.G.M.); 2 Institute of Physical Chemistry, Polish Academy of Sciences, Kasprzaka 44/52, 01-224 Warsaw, Poland; aqayyum@ichf.edu.pl (A.Q.); jcarloscolmenares@ichf.edu.pl (J.C.C.); 3 Center for Interdisciplinary Research and Innovation (CIRI-AUTH), 57001 Thessaloniki, Greece; 4 Faculty of Engineering, Universidad Cooperativa de Colombia, Medellín 50031, Colombia; ramon.colmenaresq@campusucc.edu.co; 5 Institute of Chemistry for Life and Health Sciences, Chimie ParisTech, PSL Research University, CNRS, UMR8060, 11 Rue Pierre et Marie Curie, F-75005 Paris, France; christophe.len@chimieparistech.psl.eu

**Keywords:** carbon-based nanocatalysts, heterogeneous catalysis, photocatalysis, sonocatalysis, sonophotocatalysis, lignin hydrogenolysis, 5-hydroxymethylfurfural (5-HMF) to 2,5-diformylfuran (DFF), biomass valorization, hazardous organics remediation

## Abstract

The continuous increase of the demand in merchandise and fuels augments the need of modern approaches for the mass-production of renewable chemicals derived from abundant feedstocks, like biomass, as well as for the water and soil remediation pollution resulting from the anthropogenic discharge of organic compounds. Towards these directions and within the concept of circular (bio)economy, the development of efficient and sustainable catalytic processes is of paramount importance. Within this context, the design of novel catalysts play a key role, with carbon-based nanocatalysts (CnCs) representing one of the most promising class of materials. In this review, a wide range of CnCs utilized for biomass valorization towards valuable chemicals production, and for environmental remediation applications are summarized and discussed. Emphasis is given in particular on the catalytic production of 5-hydroxymethylfurfural (5-HMF) from cellulose or starch-rich food waste, the hydrogenolysis of lignin towards high bio-oil yields enriched predominately in alkyl and oxygenated phenolic monomers, the photocatalytic, sonocatalytic or sonophotocatalytic selective partial oxidation of 5-HMF to 2,5-diformylfuran (DFF) and the decomposition of organic pollutants in aqueous matrixes. The carbonaceous materials were utilized as stand-alone catalysts or as supports of (nano)metals are various types of activated micro/mesoporous carbons, graphene/graphite and the chemically modified counterparts like graphite oxide and reduced graphite oxide, carbon nanotubes, carbon quantum dots, graphitic carbon nitride, and fullerenes.

## 1. Introduction

Considering the continuous growth of the population, which is linked with incremental demands in fuels and products and, hence, of raw chemicals and materials, the necessity for novel, innovative, environmentally friendly, and sustainable approaches and transformation technologies is continuously increasing as well. Towards this direction, the concept of circular (bio)economy has been established over the last years, aiming towards the valorization of biomass and organic wastes as abundant feedstocks to produce valuable platforms and fine chemicals. In addition to the need for sustainable and renewable resources, the extensive use of fossil raw materials and of their derived chemicals and products in everyday life has led to unprecedented “anthropogenic environmental pollution”. Water and soil contamination, as a results of the uncontrollable discharge of organic compounds (such as pharmaceuticals, pesticides, heath care products, or dyes), is considered amongst the top emerging concerns. Either for the production of renewable/recycled chemicals or the remediation of hazardous compounds, advanced catalytic processes are employed and their efficiency depends greatly on the performance of the catalytic materials.

Carbon is one of the most abundant elements on Earth that exist in various allotropes, which are of high value to humankind. In addition to bulky structures such as diamond, amorphous carbon and charcoal, the most important nanoscaled carbon allotropes are graphene, carbon quantum dots (CQDs), single- or multi-walled carbon nanotubes (CNTs), fullerenes, carbon nano-diamondoids (CNDs), carbon nanohorns (CNHs), and carbon nano-onions (CNOs) among others [1,2,3,4,5,6,7,8]. Graphtic carbon nitride (g-C_3_N_4_) is another two-dimensional polymeric material consisting of almost equal numbers of carbon and nitrogen elements [9]. All the above nanostructures possess unique physicochemical properties such as conductivity, thermo-, chemo-, photo-, and mechanical stability, low density, high specific surface area and light absorptivity, due to their nanoscale dimensions and morphology, and their structural and electronic properties (hybridization of carbon atoms, aromaticity, etc.). Another important aspect is the existing ability to tune their surface functionality on demand by chemical modifications, leading to materials with specific and desired properties [10]. Activated porous carbons or biochar (APCs), although not considered as nanomaterials, have attracted research interest and have reached wide commercialization in the fields of adsorbent and catalyst manufacturing. The properties of APCs that make them ideal for such applications are their tunable surface chemistry, the high surface area and pore volume induced by the nanosized micro/mesopores, and their exceptional chemical and dimensional stability in aqueous or various organic media [11,12,13,14]. In general, the APCs can be obtained via various synthetic approaches/routes. The two most well-known routes are with physical activation and chemical activation [15,16]. In the former, the precursors undergo carbonization at inert atmosphere in a temperature range of 500 to 800 °C in order to obtain the carbon/biochar, which is further activated by gasification in CO_2_, steam or O_2_ atmosphere [15,17]. In the case of chemical activation, the main part of the synthesis involves mixing with an activation agent and thermal treatment in inert atmosphere. The most widely used activation agents are strong acids like H_3_PO_4_, strong bases like NaOH and KOH, or salts such as ZnCl_2_ and FeCl_3_ [15,18]. Modern and more sustainable synthesis processes have been developed in recent years, such as hydrothermal or microwave-assisted carbonization/activation [18,19]. Another key aspect of APCs is that they can be obtained from residual biomass or other organic wastes (e.g., plastics, spent tires, etc.) via initial formation of (bio)char and further thermo-chemical activation [20,21,22,23].

With regard to the design of novel catalysts, most of the above mentioned nano-scaled carbon allotropes were found suitable to be utilized either as stand-alone catalysts or as substrates/supports towards the synthesis of (nano)composite/hybrid catalysts, depending on the anticipated properties and field of application. In the case of (nano)composites/hybrids, the unique features and the elevated catalytic efficiency can be related to the homogeneous dispersion of the metal active catalytic sites and the arisen synergistic effects with the carbon supports/substrates. In this review article, our focus is concentrated on presenting the utilization of carbon-based nanocatalysts (CnCs) for catalytic biomass valorization and environmental remediation applications. Special emphasis is given on the catalytic hydrogenolysis of lignin towards value-added phenolic compounds and the production of 5-hydroxymethylfurfural (HMF or 5-HMF) from cellulose and food wastes (rich in starch). Last but not least, the recent trends of utilizing CnCs for photo-, sono- and sonophoto-catalytic selective partial oxidation of HMF to 2,5-diformylfuran (DFF) and for the decontamination of hazardous organic compounds in (waste)water matrixes are discussed.

## 2. Carbon-Based Nanocatalysts for the Production of 5-HMF from Cellulose and Starch-Rich Food Waste

**Figure 1:**
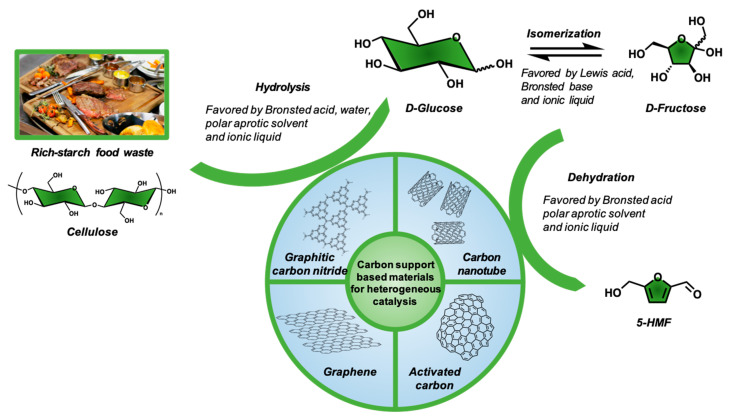
Chemical pathways for 5-HMF production from rich-starch food waste and cellulose using carbon support-based materials.

Nowadays, modern civilization has created an increasing demand for basic functionalized organic compounds for the fine chemical industry and the energy sector. Alongside this demand, the planet is faced with an awareness of climate change, green gas emissions and a rapidly shrinking fossil fuel pool. To slow down this process, several avenues exist, including the intensive production of biorenewable resources. Chemical companies are converting renewable bioresources into bulk commodities and especially to chemicals. Among a wide range of platform molecules of interest, 5-hydroxymethylfurfural (5-HMF or HMF) is considered as a platform chemical of high importance due to its various industrial applications [24,25,26,27], and is traditionally manufactured from plant waste. In general, 5-HMF is derived from cellulose through a series of chemical processes: (i) hydrolysis of the β-1,4 bonds of the anhydrous glucose unit; (ii) in situ isomerization of glucose to fructose and (iii) dehydration of fructose. Among the different technologies used for the production of 5-HMF, heterogeneous acidic catalysis, homogeneous acid catalysis, enzymatic catalysis and autocatalysis are the main processes. To date, various parameters are well known to improve the yield and selectivity of 5-HMF; among them, the nature of the catalyst (Brönsted acid, Lewis acid, Brönsted base), the strength of the acid and the synergies between catalytic sites and biphasic solvent systems to lessen the formation of by-products such as humins. Recently, modified carbon-based support materials have shown notable efficiency in the catalytic production of 5-HMF starting from either fructose, glucose, cellobiose or cellulose. Various carbon-based materials, such as activated carbon [28,29,30], carbon nanotube [31,32], graphitic carbon nitride [33,34] and graphene [35,36], have been developed and used in heterogeneous catalysis for the production of 5-HMF starting from carbohydrate moiety such as glucose and fructose. These carbon-based materials have several advantages, such as good thermal stability, comparatively higher stability in acidic or basic media, tunable hydrophobicity and polarity, easy recovery from the reaction mixture, and cost-effectiveness compared to other conventional catalysts. Unfortunately, the use of cellulose and wastes has been poorly reported despite the fact that only these substrates will allow an efficient and competitive production of 5-HMF as platform molecule. For the sake of clarity, this chapter focuses on the recent state of the art in the field of catalytic production of 5-HMF from cellulose and starch-rich food waste in the presence of modified carbon support based materials (Figure 1).

### 2.1. Activated Carbon Based Catalysts and Derivatives

Biochar, charcoal and activated carbon are three very similar forms of carbon, with very similar composition and production methods. After functionalization with Brönsted acidic groups the presence of acidic oxygen functional groups such as SO_3_H, PO_3_H and -COOH on the surface of mesoporous carbon seems to be beneficial for the adsorption of β-1,4 glucan chains, which may help in the disintegration of the cellulose network. The main works on the production of 5-HMF from cellulose and starch-rich food waste focus to the use of activated carbon-based materials. Azar et al. reported the formation of 5-HMF using modified mesoporous commercial carbon of trade name SA-30 [37]. Among different acidic catalysts, the authors reported the oxidative treatment of SA-30 with a saturated solution of (NH_4_)_2_S_2_O_8_ in aqueous H_2_SO_4_ (1 M) at room temperature for 1 day with the obtained material to be referred to as SAS carbon. The treatment of commercial microcrystalline cellulose (500 mg) in the presence of the unmodified SA-30 (125 mg) in distilled water (25 mL) at 190 °C for 3 h under pressure gave a cellulose conversion of 56% and a 5-HMF selectivity of 28%. On the other hand, the use of SAS catalyst having Brönsted acidic groups led a cellulose conversion of 60% and a 5-HMF selectivity of 22%. SA and SAS catalysts gave a glucose selectivity of 35% and 52%, respectively. Consequently, the surface functionalization with SO_3_H group was permitted to have a low-cost modified carbon material, SAS, which combines the required acidity and suitable textural material properties to gain glucose with good selectivity (52%). However, SAS was not efficient for the dehydration of glucose to 5-HMF. This can be explained either by a limit in the positive effect of acidic OFG, by the lower surface area and pore volume, or by a combination of these two reasons. The characterization showed that SA-30 activated carbon has a high specific surface area (1464 m^2^ g^−1^) and well-developed porosity in the micro and mesopore range (V_micro_ = 0.74 cm^3^ g^−1^ and V_meso_ = 0.73 cm^3^ g^−1^). On the other hand, SAS with sulfonated moiety revealed lower values for the textural features (specific surface area 1274 m^2^ g^−1^; micropore volume 0.56 cm^3^ g^−1^ and mesopore volume 0.48 cm^3^ g^−1^), which may be linked either to pores destruction or/and to blockage due to the development of the surface oxygen groups. It is noteworthy that the conversion of cellulose to 5-HMF was conducted in water, as sole solvent, and other chemicals were also obtained but no more analysis was reported concerning the nature of the mixtures. The strategy to use water did not permit the effective extraction of the target compound and resulted in the production of different polymers due to condensation reactions.

Sulfonated biochar (SBC) was also used for the production of 5-HMF from food waste. In this sense, Cao et al. described the production of 5-HMF starting from starch-rich food waste such as bread generated at Hong Kong International Airport [38]. Biochar was prepared from forestry wood waste by slow pyrolysis up to 700 °C for 15 h and then grounded and sieved through a 0.25-mm mesh. The resulting biochar (10 g) in the presence of concentrated H_2_SO_4_ (150 mL) was mixed at 150 °C for 12 h and SBC was obtained after washing and drying. Biochar and SBC have different specific surface areas (135.2 m^2^ g^−1^ vs. 6.90 m^2^ g^−1^) and the strongly acidic –SO_3_H group amounted to 40% of the total acidity density. In order to have a better conversion, microwave-assisted food waste transformation was studied for the production of 5-HMF. This innovative technology has many advantages: energy efficiency, safer solvents, material reusability, catalysis, fewer steps, increases product yield and/or selectivity [39]. To optimize the production of 5-HMF, a biphasic system of an organic solvent and water was studied extensively for glucose/fructose dehydration in order to limit further condensation reactions of 5-HMF. Bread waste (0.5 g) in a mixture of DMSO-H_2_O (3:1, *v*/*v*) in the presence of SBC (0.5 g) at 180 °C for 20 min led to a 30% HMF yield. With regeneration process, SBC displayed excellent recyclability for comparable HMF yield from bread waste over five cycles (5-HMF yield 30% for the first cycle vs. 26% for the fourth cycle). The same group reported a similar work by using of phosphorylated biochars to catalyze the conversion of starch-rich food waste such as bread, rice and spaghetti [40]. After convention treatment of pinewood sawdust and chemical activation with H_3_PO_4_ at 400–600 °C, different phosphorylated carbons/biochars were produced in a single step. High activation temperature and impregnation ratios permitted to have a high specific surface area (1547 m^2^ g^−1^) and a well-developed porous structure with favorable meso-porosity (micropore volume 0.51 cm^3^ g^−1^ and mesopore volume 0.66 cm^3^ g^−1^). Microwave-assisted food waste transformation was studied for the production of 5-HMF. Food waste (0.5 g) in a mixture of DMSO-H_2_O (3:1, *v*/*v*) in the presence of phosphorylated biochar (0.2 g) at 180 °C and 15 bar for 20 min formed 5-HMF in a yield of 30%. Keeping the same reaction conditions, to obtain similar results the weight of phosphorylated biochar was lower than that of sulfonated biochar (0.2 g vs. 0.5 g). Having sulfonated or phosphonated groups, the productivities were similar.

Another approach developed for the formation of 5-HMF was based on the combination of modified carbonaceous materials and ionic liquids. Ionic liquids are considered as important solvents because the insolubility of cellulose in most of the common organic solvents and water limits the efficiency of the process. Tyagi et al. reported the conversion of microcrystalline cellulose to 5-HMF using the association of modified activated carbon and commercial 1-butyl-3-methylimidazolium chloride [BMIM]Cl as ionic liquid [41]. After the treatment of activated carbon (20 mg) with H_2_SO_4_ (18 M, 100 mL) for 4 h at 75–80 °C followed by conventional work-up, sulfonated activated carbon (AC-S) was obtained. Compared with the parent activated carbon, the AC-S catalyst has a more rough and porous structure with a surface area of 780 m^2^ g^−1^, a total volume of AC-S was (2.57 cm^3^ g^−1^) with an average pore size of ~0.9 nm. Starting from microcrystalline cellulose (100 mg) in [BMIM]Cl (2 g) in the presence of SAC (50 mg) at 120 °C for 12 h, 5-HMF was obtained in 33% yield. HMF yield was decreased (4% vs. 33%) after 8 recyclings, suggesting the loss of ionic liquid after each cycle with the existing amount not to be sufficient after different recycling. The addition of water and immobilization of a metal, such as Cr^3+^, improved the process, and hence this strategy can be considered for further studies. The authors reported that the addition of ionic liquid increases the product yield and decreases the reaction time significantly. This indicates that the addition of [BMIM]Cl thermodynamically favors the recalcitrant breaking of cellulose, leading to fast breakage of β-1, 4 glycosidic bonds. Nevertheless, ionic liquids are not assumed as green solvents, since recent works have shown that ionic liquids can be toxic and hazardous as well [42,43].

The association of sulfonated carbon materials and ionic liquids was reported to obtain the advantages of both. In this context, Zhang et al. described the production of 5-HMF in water starting from cellulose in the presence of fluorine anion-containing ionic liquid and modified with sulfonic acids biochar [44]. The catalyst was prepared in four steps: (i) biochar sulfonic acid (BCSA) was obtained by mixing successively carbonized bamboo powder with aqueous H_2_SO_4_ (80%) and oleum (SO_3_ 50 wt%); (ii) after different treatments using NaCl and ultrasounds, BCSA was transformed to BCSANa powder; (iii) the powder was treated with IL-Cl for 12 h to furnish BCSANa-IL-Cl and (iv) the chloro-derivative was then acidified by an excess of CF_3_SO_3_H at room temperature for 1 day, washed with hot water and dried at 120 °C for 12 h. The density of SO_3_H groups on BCSA-IL-F was 1.31 mmol g^−1^, while introduction of IL-F improved the stability of SO_3_H groups. Treatment of microcrystalline cellulose (0.2 g) catalyzed by BCSA-IL-F (0.1 g) in pure water at 80 °C under microwave irradiation produced 5-HMF in 28% yield with a TON of 4.89. The process presented many advantages: (i) cheap and available BCSA-IL-F as heterogeneous catalyst and water as solvent; (ii) mild, easily and green operating conditions and (iii) BCSA-IL-F shows a high activity and good selectivity for 5-HMF. After 6 cycles, the yield of HMF is similar to that obtained with fresh catalyst BCSA-IL-F catalyst, indicating that the catalyst is stable and has good repeatability.

Instead of grafting SO_3_H and PO_3_H groups, different metals were supported on carbon materials to increase the number of Lewis acid groups. Liu et al. reported the use of corn stalk as raw material for the production of 5-HMF [45]. Powdered corn stalk was mixed with a solution of MgCl_2_/SnCl_4_ (0.5 M) using ultrasound radiation and heated for 5 h at 105 °C. The magnesium and tin ion-loaded biochar (Biochar-Mg-Sn, 10:1 wt:wt) was found to have a Ca^2+^, Mg^2+^ and Sn^4+^ weight ratio of 5.09, 50.17 and 33.10 mg per gram, respectively. Its surface area was 104 m^2^ g^−1^ and its pore average diameter was 1.80 nm. Corn stalk in the presence of biochar-Mg-Sn (20%) in isopropanol/1-allyl-3-methylimidazolium chloride (AMIMCl) at 100 °C for 3 h showed 63% conversion to 5-HMF with a selectivity of 69%. The authors reported that the conversion of corn stalk to 5-HMF is mainly catalyzed by Sn located on the biochar. Tetra-coordinated Sn could convert biomass into fructose and 5-HMF, successively. Association of biochar-Mg-Sn and isopropanol/AMIMCl arisen synergistic effects resulted in an increase of the conversion. The reusability of the biochar-Mg-Sn catalyst was also studied, and the 5-HMF yield was decreased by only 15% after five recyclings.

Yu et al. reported the production of 5-HMF starting from bread as starch-rich food waste in the presence of SnCl_4_ as homogeneous catalyst [46]. The authors mentioned that polymerization-induced metal-impregnated high-porosity carbon was a possible precursor of biochar-based catalyst further driving up the economic potential.

### 2.2. Carbon Nanotubes (CNTs) Based Catalysts

One interesting allotrope of carbon, which has already utilized in a plethora of applications since its discovery in 1991 (credited to Sumio Iijima), is carbon nanotube [47]. This one-dimensional form consists of a rolled-up single-layer of carbon atoms, namely graphene, forming one dimensional tube-like nanostructures with a diameter ranging in the nanoscale and a length from several micrometers up to few millimeters. If carbon nanotube (CNT) is made from one layer, it is called single-walled (SWCNT), while if more layers are involved, multi-walled (MWCNT). Due to their unique nanostructure as well as the sp^2^ hybridization of the bonds between carbon atoms and the arisen geometry of the hexagonal/pentagonal carbon-atom lattices (pitch of the helicity), CNTs possess attractive and tunable physicochemical properties, while they can act either as metal by being totally electrically conductive, as semi-conductors, or either as insulators (non-conducting) [48].

Carbon nanotubes (CNTs) were also used for the production of 5-HMF due to their excellent mechanical, physical and chemical properties. CNTs-based materials have rarely been used as catalysts to convert cellulose. On the other hands, CNTs were used as mass-transfer promoters in the presence of homogeneous acid catalysts [49]. Microcrystalline cellulose (5.83 g) in the presence of homogeneous catalyst HCl (200 ppm) and carbon nanotube (CNT, 386 ppm) were mixed in a solution of methyl-isobutyl-ketone (MIBK, 218 mL) and water (175 mL) at 140 °C and 10 bar for 2 h. An increased cellulose conversion of 43% was achieved, with the CNTs to play a key role since the conversion in their absence was only 20%. It is obvious that CNTs improved the cellulose transformation into 5-HMF in a water-MIBK biphasic system by 5-HMF liquid mass transfer. The authors reported that CNT was a promising mass-transfer promoter, enhancing the extraction kinetic more than 3.7 times, mainly under acid conditions. As several equilibria steps are involved in the process, this extraction displaced all the reaction, also yielding to an increase in the productivity (270 times higher). In the same work, Faba et al. also tested activated carbon as a mass-transfer promoter, although its efficiency was found negligible [49].

### 2.3. Graphene Based Catalysts

Graphene is an allotrope of carbon, the thinnest two-dimensional (2D) nanomaterial with a thickness of one carbon atom. It consists of sp^2^ hybridized carbons organized in a honeycomb lattice, and hence the structure is rich in delocalized electrons. Even though graphene as an allotrope was proposed theoretical in 1947 [50], it was the groundbreaking work of Geim and Novoselov in which was presented the successful separation and identification of graphene by mechanical separation using Scotch tape, with the authors winning a Nobel Prize (2010) for this work [7,51]. Due to its unique properties, including supreme chemical, thermal and mechanical stability, optical transparency, high electrical and thermal conductivity, electron mobility and the great specific surface area per weight (2630 m^2^/g), graphene has already been used in a plethora of materials for various applications [52]. Chemical modification/functionalization of graphene with different heteroatoms/groups is feasible and can arise new on-demand properties. In general, modification of graphite can be achieved by the introduction of functional groups/atoms covalently bonded to the structure. Another way of modification is the introduction of chemical moieties/functionalities with a non-covalent nature of interactions, such as hydrogen-bonding, π-π or electrostatic interactions [53]. Although, the non-covalent approach has the advantage of maintaining the chemical and physical properties of graphite unchanged, chemical modification leading to covalent in nature incorporation of surface functionalities is predominately used for the design of materials utilized for the herein presented catalytic applications. For instance, oxidation can lead to the incorporation of oxygen-containing functional groups, such as epoxy, carbonyl, hydroxyl and carboxyl [54,55], and thus the interlayer distance of the sheets can be almost double (from ~0.34 nm to ~0.65 nm). The oxidized form of graphene is referred to as graphene oxide (GO) while reduction resulting in reduced graphene oxide (rGO), with the oxygen containing functionalities to be almost disappeared and defects to be formed due to carbon atoms elimination from the lattice [56,57].

Graphene and its chemical modified counterparts, especially graphene oxide (GO) and reduced graphene oxide (rGO), have attracted the attention of the scientific community as a new and emerging carbon nanomaterial having excellent physical and chemical properties, such as good thermal conductivity, large surface area, etc. Sulfonated graphene quantum dots (SGQDs) were developed as quasi-homogeneous catalyst for the production of 5-HMF from cellulose at high concentration [58]. In order to prepare SGQD, graphene quantum dots (GQDs, 1 g) were calcinated under N_2_ at 300 °C for 1 h and then treated with chlorosulfonic acid (5 mL) in chloroform at 70 °C for 1 day. Conventional wash and dry led to the catalyst powder SGQD by a facile practical synthesis. Ball-milled cellulose (25 mg) and SGQDs (100 mg) in a mixture of DMSO-water (7:3, *v*/*v*) as biphasic solvent system at 170 °C for 2 h afforded 5-HMF in 22% yield with a selectivity of 28%. The authors reported that SGQDs combine the merits of homogeneous and heterogeneous catalysts. DFT calculations showed that intra-molecular hydrogen bonded -OH groups and the adjacent -SO_3_H groups on SGQDs play a synergistic role in their high catalytic efficiency. SGQDs showed good efficiency and recyclability in the dehydration reaction of fructose (5-HMF yield 50% for the first cycle vs. 40% for the seventh cycle). Unfortunately, the recovery of SGQDs was not reported starting from ball-milled cellulose.

To our knowledge, the yield and selectivity of 5-HMF can be increased by optimizing the following parameters: the nature of the catalysts (Brönsted acid, Lewis acid, Brönsted base), the strength of the acid and the synergies between the catalytic sites and the two-phase solvent systems to decrease the formation of by-products such as humins.

Since this chapter summarizes the recent advances for the synthesis of 5-HMF starting from cellulose and rich-starch food waste using modified carbon-based materials, the nature of catalysts was the guiding criteria taken into account. In general, sulfonated [37,38,41,44,58] and phosphonated catalysts [40] were developed as Brönsted acid heterogeneous catalysts using activated carbon [37,41], biochar [38,40,44] and graphene [58], even if metals having Lewis acid properties have been reported [45,46]. The most common processes used biphasic systems [38,40,49,58] to decrease the contact between 5-HMF and carbohydrate and therefore to decrease the formation of humins. Nevertheless, water as sole solvent can lead to the formation 5-HMF [37]. Commercial ionic liquids and their derivatives have been successfully tested for the production of 5-HMF; however, the toxicity of these solvents and their cost need to be addressed [41,44,45]. It is noteworthy that CnCs have been reported as mass promoter in the presence of homogeneous catalyst [49]. In parallel with convention heating, microwave irradiations were also proposed as a prosperous process intensification tool in terms of activation [38,40,44]. The application of modified carbon-based materials shows great promise for the catalytic formation of 5-HMF from cellulose and food waste, although the latter is not widely used. The same process to continuous flow, which has not been studied to date, is an approach which can open new horizons towards more cost-effective processes that could achieve the goal of scale-up.

## 3. Photo- and Sono-Catalytic Selective Oxidation of HMF and Decomposition of Organic Pollutants

Photocatalysis has attracted increased attention in recent decades as one sustainable and “green” efficient approach for a plethora of applications, with the ultimate goal to use one of the most abundant energy sources, sunlight. Intense emphasis is given for utilization of photo-assisted catalytic methods for facing environmental organic pollutants as, for instance, pharmaceuticals, dyes, detergents, volatile organic compounds, etc. In the heterogeneous photocatalytic processes, oxidation and reduction reactions predominately take place on the materials’ surface, because of the formation of photo-induced electron and hole pairs upon exposure to light, leading to the generation of free radicals such as hydroxyl radical (OH•), hydroperoxyl radical (HOO•) and superoxide radical (O_2_−•), which are responsible for the un-selective degradation of organic pollutants in aqueous matrixes [59].

Ultrasound irradiation as a source of power for catalytic applications, namely sonocatalysis, also gathers research attention as a promising technique/approach due to physical and chemical effects, which are presented as a result of cavitation phenomena upon ultrasonication of an aqueous media [60]. Cavitation phenomenon is a process of adiabatic generation, growth and collapse of microbubbles leading to the formation of the known “hot spots” with the temperature and pressure reaching up to 5000 K and 500 atm, respectively [61]. The elevated temperature and pressure in combination with the formation of free radicals (H•, OH•, and O_2_−•) resulted predominately from the homolytic fission of water, establishing the area close to hot spots as “nano-reactors” of high activity [62]. In addition, the creations of the hot spots are responsible for other physical/mechanical effects (shock waves, mass transfer, microjets, shear forces, and de-aggregation of (nano)particles), which are important in cases of heterogeneous catalysis [61].

The combination of sonocatalysis and photocatalysis is known as sonophotocatalysis or photosonocatalysis. This combined catalytic technique is found to be very attractive lately for converting the pollutants to less hazardous components/products due to its cost-effective and environmental process [63]. The combination of these two sources of power can overcome various limitations and synergistically increase the efficiency and selectivity of the catalytic processes [61,62]. Even though for environmental remediation applications the un-selective decomposition/mineralization of the organics is desirable, when the use of the catalytic method is for synthetic chemistry, where selectivity matters the most, the control/manipulation of photo- or/and sono-reactivity is a key feature.

To achieve the anticipated results in catalytic processes, the design and development of the appropriate catalyst is vital. Hence, in this part, we present and discuss various carbon-based nanomaterials (CnCs) that have been explored and presented advanced photocatalytic, sonocatalytic, or/and sonophotocatalytic for both unselective decomposition of organic compounds, which are assumed as pollutants. In Table 1 are collected characteristic CnCs materials used for the sono- and/or sono-photo-catalytic decomposition of organic pollutants. In addition, the selective partial oxidation of 5-HMF to DFF is presented and discussed in the frame of biomass valorization, since the predominant research weight is focused on this reaction.

### 3.1. Carbon Nanotubes (CNTs) Based Catalysts

In the case of nanocomposite consisting of carbon nanotubes (CNTs) with semiconductor phases/materials, the development of synergistic effects has been observed having a positive impact on the degradation of organic pollutants, predominately by enhancing the separation lifetime of the e^−^/h^+^ pairs (created due to the presence of the semiconductor photoactive phase), while the formed Schottky junction at the interface of the two phases helps on the electrons or/and holes delocalization [76]. Moreover, the formation of bonds between the carbonaceous phase with the inorganic phase, as for instance TiO_2_, can result in a narrowing of the energy difference between the covalence and valence band (band gap) or the generation of intermediate energetic states. Additionally, the incorporation of nanoscaled CNTs promotes the better dispersion of the active catalytic sites and helps also upon the synthesis of the inorganic materials/composites by increase the nucleation sites.

**Figure 2:**
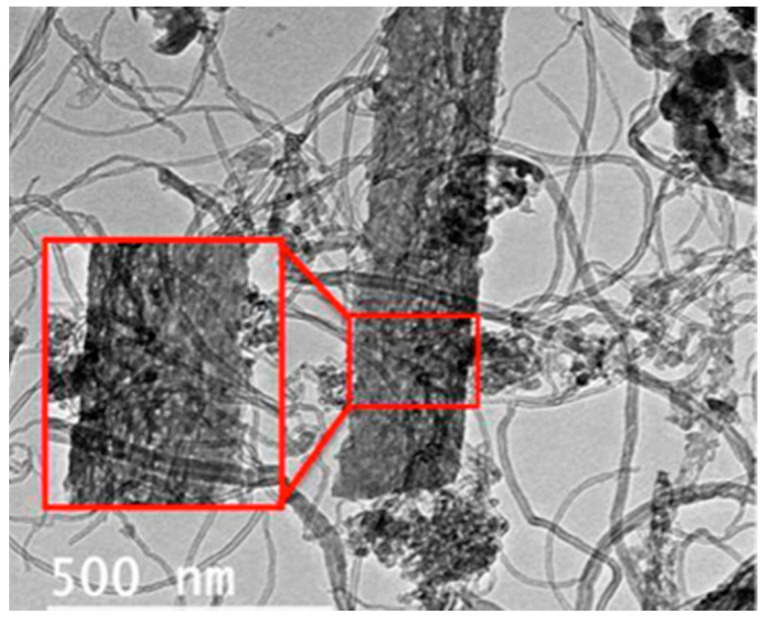
TEM micrograph of an intergrowth of carbon nanotubes with TiO_2_-B nanoribbons. Reprinted with permission from Ref. [76]. Copyright 2018, Springer Nature.

For instance, Shaban et al. studied the photocatalytic degradation of methylene blue (MB) dye by using a composite of CNTs and TiO_2_ nanoribbons (TiO_2_ NRs/CNTs, Figure 2) under sunlight and the results showed that TiO_2_ NRs/CNTs possessed 12% more photocatalytic efficiency as compared to TiO_2_ nanoribbons [76]. Chen et al. [77] also studied the photocatalytic degradation of Rhodamine-B (Rh-B) by using CNTs/TiO_2_ under sunlight irradiation. It was found that the CNTs/TiO_2_ exhibited a 50% higher photocatalytic degradation efficiency against Rh-B as compared to pure TiO_2_. The authors linked this photocatalytic enhancement to the formation of n-n type heterojunctions, which promote the transportation and migration of photogenerated charge carrier, with their delocalization to promote reactions with H_2_O and O_2_ in order to form hydroxyl and peroxide free radicals, respectively. The design of CNTs-based photocatalyst with other active phases rather than TiO_2_ is feasible. For example, Phin et al. [78] reported that ZnO/CNTs composite possesses higher degradation efficiency against methylene blue (MB) dye than the pure ZnO under sunlight irradiation. This was linked by the authors to the increase of the band gap energy with a simultaneous blue shift upon composite formation. The ZnO nanoparticles were attached on the CNTs via zinc carboxylate groups, while 10 wt.% of CNTs was found to be the optimum amount regarding the photocatalytic performance.

Due to their nanoscaled structure and the unique chemical features, CNTs were found as prosperous ultrasound catalyst even utilized solely. Al-Hamadani et al. [64] presented that the sonocatalytic decomposition of two pharmaceuticals products, sulfamethoxazole and ibuprofen, was elevated significantly in the presence of CNTs at a frequency of 1000 kHz. It is worth mentioning that ultrasound irradiation plays a determinate role in liquid/solid heterogeneous catalysis by improving the degradation extent in addition to the increase of the system’s temperature and the dispersion/de-aggregation of the (nano)particles. The sonocatalytic ability of more complex materials was studied by Li and co-workers [65]. They studied the sono- and sonophoto-catalytic efficiency of TiO_2_ nanoparticles decorated on CNTs, with or without carbon quantum dots (CQDs) doping. The latter material, referred to as CQDs/CNTs-TiO_2_, showed a superior degradation performance against Rh-B compared to TiO_2_/CNTs both under 20 and 30 kHz frequency. CQDs/CNTs-TiO_2_ showed enhanced Rh-B adsorption ability, as well as light absorption, leading to the creation of e^−^ and h+ upon photoexcitation which were transferred to the conduction and valance band of TiO_2_ and CQDs, respectively. Moreover, in the presence of CNTs, the transfer of photogenerated species to the surface of catalyst increases. Ahmad and co-workers showed also that the combination of ultrasound and light as sources of power simultaneously led to elevation of kinetics and removal efficiency of Rhodamine B, compared to photocatalysis or sonocatalysis separately, when a ZnO/CNTs was used [79]. This enhancement can be linked to the arisen synergistic effects upon combination of the two sources, as it was also showed in the case of selective oxidation of monoaromatics in the presence of a photocatalyst [61].

Functionalized CNTs-based materials have been explored for biomass valorization only thermo-catalytically. For instance Zhou et al. [80] reported the decoration of noble metals’ (Pt, Au, Pd, Ru and Ir) nanoclusters on CNTs following a microwave-assisted “ethylene glycol” reduction method, and the composites materials were tested for the thermo-catalytic aerobic oxidation of 5-hydroxymethylfurfural (HMF) in water under base free condition. Pt/CNTs showed the highest HMF conversion (100%) among all the other nanocomposite and good selectivity towards 2,5-furandicarboxylicacid (FDCA). Even more interesting, Pt nanoparticles were decorated on various other supports like SiO_2_, Al_2_O_3_, ZrO_2_, graphite oxide, activated carbon, and still the use of CNTs was found to be the most efficient for the aerobic oxidation of HMF. The formation of FDCA was linked to the presence of the oxygen-containing functional groups such as phenol, quinone and carbonyl on the surface of CNTs. These functional groups act as adsorption centers for HMF and facilitate further the required hydrogen and oxygen transfers.

### 3.2. Carbon Quantum Dots (CQDs) Based Catalysts

Carbon quantum dots (CQDs) are metal-free carbon-based fluorescent nanomaterials with size of few nanometers. In general, CQDs consist of a pure carbon core, which is surrounded by an amorphous phase that can possesses different surface functional groups like carboxyl, hydroxyl, epoxy, amides, amino, etc. [81]. The chemical heterogeneity as well as the size of the CQDs depends on the precursors and the synthesis method, which can be either top-down or bottom-up [82,83]. Among the various potential precursors, intense research attention is focused on utilizing natural resources or/and biomass residues/waste for the synthesis of CQDs, such as lignocellulosic residues, agricultural or food wastes [84,85]. By modifying the surface chemistry and tuning of the size, depending on the synthetic route and precursors, it is possible to engineer the band gap, light absorptivity, and surface reactivity and hence CQDs have an expanded range of applications. Due to their ability to harvest light from ultraviolent and up to infrared region of the electromagnetic range, CQDs were widely used for photocatalytic applications [86,87].

CQDs-based composites are used broadly as metal-free materials for environmental remediation applications owing to low fabrication cost, high water dispensability, high chemical stability, and excellent photostability. Hu et al. [88] established the mechanism of CQDs usage as a pure photocatalyst under visible-light irradiation and discovered the important factors influencing the photocatalytic activities of CQDs linked to the presence of specific oxygenated functional groups on the surface of CQDs. It was also observed that carbonyl (C=O) and a carboxyl (-COOH) groups might induce the high upward band bending due to which the recombination of photogenerated electron/hole pairs could be inhibited and hence enhanced photocatalytic efficiency to be achieved. The authors also showed that CQDs possess an elevated photocatalytic efficiency against methylene blue (MB) under visible-light irradiation, with CQDs’ size and surface chemistry to play a key role.

In the recent era, different kinds of photocatalytic mechanisms have been proposed for CQDs based catalysts. Generally, CQDs-based photodegradation of organic pollutants involves two stages. Firstly, the absorption of light, which leads to the generation of electrons/holes pairs. Secondly, the inhibition of photogenerated electrons holes pairs recombination, which leads to the formation of active species that proceed with the photocatalytic process [89]. CQDs have electron-accepting and delocalization property, and hence inhibit the photogenerated electron/hole pairs. In the case of composites of CQDs with other semiconductor metals/metal oxides, the lifetime of the photoexcited species formed at CQDs is prolonged due to their transfer to the other phase, boosting the photocatalytic reactivity upon the formation of active reactive oxygen species.

Huang et al. [90] have reported the photocatalytic degradation of Rh-B under simulated sunlight by using Bi_3_O_4_Br nanosheets and CQDs modified Bi_3_O_4_Br (CQDs/Bi_3_O_4_Br) nanosheets. The results showed that the photocatalytic degradation activity of CQDs/Bi_3_O_4_Br nanosheets was up to four times higher compared to pure Bi_3_O_4_Br nanosheets. This was linked to the formation of the heterojunction structure between CQDs and Bi_3_O_4_Br, leading to enhanced separation of the photogenerated charge carriers. Semiconductor phases (e.g., TiO_2_, Bi_2_O_3_, ZnO, Cu_2_O and Fe_2_O_3_) in combination with CQDs towards nanocomposites formation was considered also as a promising materials’ design approach. For example, Hazarika and Karak [91] studied the photocatalytic activity of TiO_2_ and CQDs/TiO_2_ composite for the degradation of organic monoaromatic pollutant such as phenol and benzene under sunlight. The composite has showed significantly enhanced photocatalytic decomposition activity against both monoaromatics, with the authors linking this to the enhanced electron–hole separation.

In another work by Shen and co-workers, CQDs were synthesized by a hydrothermal method using as precursors glucose or citric acid and they were utilized to form composite with commercial TiO_2_ P25 [92]. It was shown that both precursors led to CQDs of uniform spherical size, although the derived CQDs from citric acid (CQDs-ca) had a size in the range of 2–4 nm, while the glucose-derived dots (CQDs-g) were slightly bigger in size (3–6 nm). CQDs-g showed a better degree of graphitization compared to CQDs-ca, and the authors linked this to the mechanism of formation, with the formation of HMF from glucose prior to the carbonization step (dehydration) to play a key role, an aspect that is important upon designing materials derived, for instance, from the formed humins during biomass valorization.

**Figure 3:**
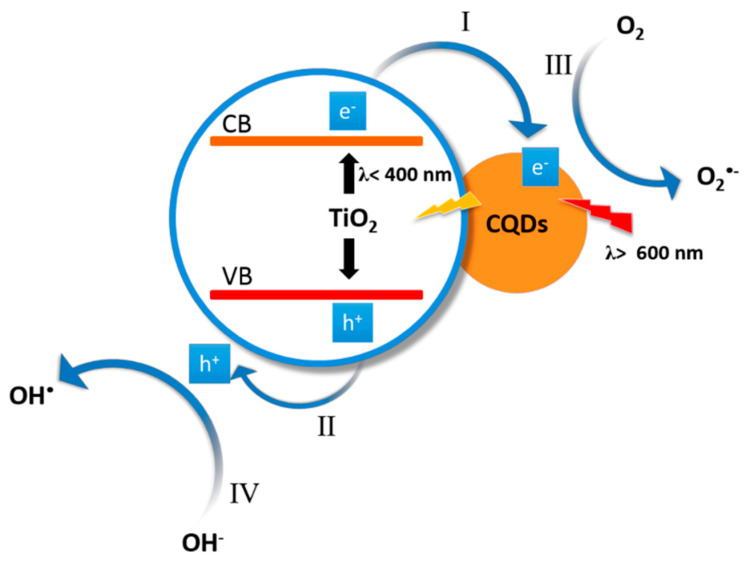
The involved phenomena and mechanisms linked to the photocatalytic activity of composite of TiO_2_ with carbon quantum dots (CQDs) under visible light irradiation. Reprinted with permission from Ref. [93]. Copyright 2016, Elsevier.

Composites of mesoporous TiO_2_ and different amounts of CQDs were also synthesized based on sol-gel and ultrasonic-hydrothermal method and studied as photocatalysts against methylene blue photodecomposition [93]. The incorporation of CQDs had a positive impact on the photoreactivity under visible light irradiation. The greatest photodecomposition activity was revealed for the composite with 5% of CQDs, which removed 98% of the dye within one hour. whilst the commercial TiO_2_ P25 showed only a 10% removal. The addition of CQDs led to synergistic effects due to composite formation enabling the photoreactivity under visible light. CQDs can absorb long-wavelength light within the visible range and emit back light with a wavelength of ultraviolent range, and hence excite the semiconductor inorganic phase to form photogenerated holes and electrons. CQDs also act as electron reservoirs by entrapping the photogenerated electrons at the conduction band of TiO_2_ phase and, in addition, hindering the recombination of photoexcited electrons and holes. The authors also studied the involved mechanisms by various means such as by degassing of the solvent (N_2_ purging) or using a colorless organic molecule (N-benzylideneaniline) and the potential of methylene blue to act as photosensitizers (sensitization mechanism) was excluded. The removal of the dissolved oxygen resulted in significantly lower photoreactivity, and so it can be concluded that superoxide anion radicals were the active species together with the formed hydroxyl radicals. All the involved phenomena and mechanisms linked to the photocatalytic activity of the composite under visible light irradiation are illustrated in Figure 3.

Composite of mesoporous TiO_2_ with CQDs (1.2 nm average size) were prepared by Olmos-Moya et al. [94], using a microwave-based approach and orange peels as CQDs’ precursor. The synthesized composites showed a red-shift of the band gap (2.08–2.30 eV) with their efficiency to convert the ultraviolet light to current upon utilization as photoanodes. Zhang et al. fabricated composites of titanium dioxide with CQDs by impregnation at room temperature, with the dots derived from coal tar pitch [95]. The composite with the optimum amount of carbon dots (6 wt.%) showed by far the highest visible-light photo-assisted decomposition of rhodamine B (RhB) dye, with the rate of degradation to be 43.7 times higher comparing to pure TiO_2_. The characterizations revealed the formation of Ti-O-C bonds, which were suggested to play a role on the photogenerated electron/hole pairs, avoiding their fast recombination. The authors were able to detect the two separate phases at the composite by high-resolution TEM imaging, since the graphitic phase had a characteristic lattice fringes distance of 0.212 ± 2 nm, while TiO_2_ crystalline of 0.350 nm.

**Figure 4:**
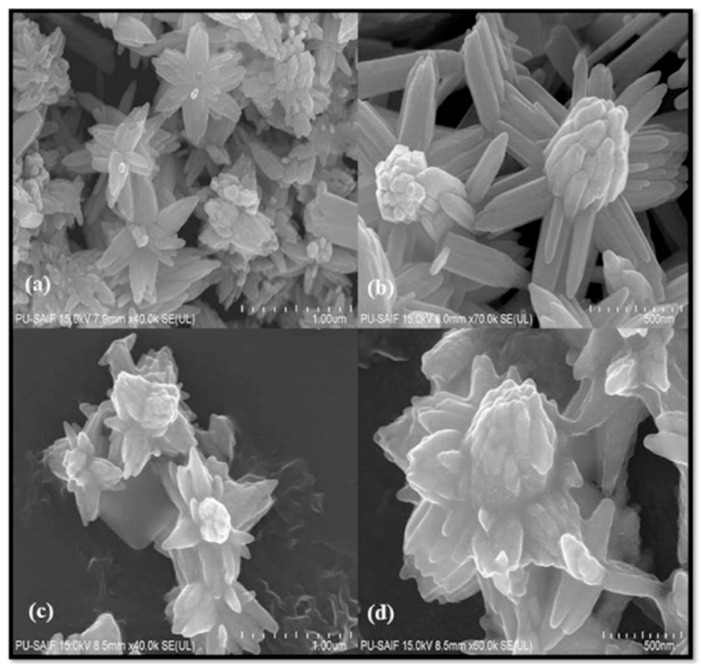
FESEM images of nitrogen doped ZnO (**a**) and (**b**) and of the N-ZnO/CQDs composites (**c**) and (**d**). Reprinted with permission from Ref. [97]. Copyrights 2017, Elsevier.

Zinc oxide composite with CQDs was synthesized based on a sol-gel followed by a spin-coating processing and tested as photocatalyst against Rhodamine B [96]. The composite showed an almost three-folds higher photo-decomposition capability compared to pure ZnO, with the enhancement to be linked to the amount/layers of CQDs, which improve the separation of the photoinduced e^−^/h^+^. Attempting to go a step further and to improve the visible light driven catalytic performance, Sharma et al. hydrothermally prepared an advantageous composite consisting of nitrogen-doped ZnO nanoflowers functionalized with CQDs (Figure 4) [97]. Nitrogen doping had a positive effect on the photodecomposition of malachite green (MG) dye under visible light, while the composite showed the highest removal both against MG, as well as against a levofloxacin drug. Mechanistic exploration using scavengers revealed that the elevated photoreactivity upon composite formation was accredited to the e-/h+ separation due to the presence of CQDs. An analogue composite was also prepared previously and showed high photocatalytic efficiency against three dyes, namely methylene blue, fluorescein, and malachite green, under daylight irradiation [98]. It is worth mentioning that the addition of CQDs also led to an enhanced anti-photocorrosion capability since the composite showed a high photocatalytic activity even for four consecutive cycles. In conclusion, the incorporation of ZnO N-doping with CQDs to synthesize a new class of photocatalysts was presented as a prosperous approach which should be applied for other metal oxides-based materials.

Functionalization of CQDs with heteroatoms like N, S, Cl, F, etc., was showed to be an efficient approach not only for catalytic applications, but also to promote the antibacterial activity, electrochemical methods, detection of specific compounds (like dopamine in human fluids), bioimaging and more [81,99,100,101]. Sulfonated graphene quantum dots (sGQDs) were found to be effective as quasi-homogeneous catalyst for the chemo-catalytically conversion of carbohydrates, such as glucose, cellulose, or fructose, to 5-hydroxymethylfurfural (HMF) [58]. This work pointed out the prosperity of using sGQDs as quasi-homogeneous catalysts for one-pot biomass valorization applications, due to their unique physicochemical properties and especially the arisen synergistic effects of the formed intra-molecular H-bonds and the -SO_3_H surface functionalities.

CQDs were also supported on Metal-Organic Frameworks (MOFs), as in particular was presented by Wang and co-workers, who used a MIL-125(Ti) framework functionalized with -NH_2_ groups as support [102]. The obtained composite with the optimum CQDs amount of 1 wt.%, referred to as CQDs/NH2-MIL-125, showed an elevated photodecomposition efficiency against PhB under different light irradiation. The CQDs not only acted as electron acceptors promoting the e−/h+ separation, but also as converters of near-infrared light to visible internally to the material.

### 3.3. Graphene-Based Nanocatalysts

The utilization of graphite oxide (GO) was shown to have a positive impact on sonocatalytic applications. For instance, Al-Hamadani et al. investigated the ultrasound assisted removal of an anti-inflammatory (diclofenac, DCF) and an anticonvulsant medication (carbamazepine, CBZ) using graphene oxide as a process intensification medium [103]. The presence of GO enhanced the decomposition of both pharmaceuticals under three studied frequencies (28, 580, and 1000 kHz). The removal of the organic contaminants was linked to sono-degradation as a result of the free radicals formation due to the cavitation as well as due to adsorption phenomena. Hence, the frequency of ultrasound had a bimodal role, both on the formation of the active oxygen containing species and on the GO sheets dispersion/exfoliation.

Although graphene or graphite (Gr) and the chemical functionalized derivatives standalone do not possess photocatalytic abilities, various forms of graphene are widely utilized either as fillers or as substrates for designing and synthesis of novel composite/hybrid materials for photo- or/and sono-catalytic driven environmental remediation applications, biomass valorization and beyond [104]. Since the amount of available worth to be mentioned articles regarding the utilization of graphene/graphite-based composites is enormous, the goal of this work was not to introduce and discuss as many as possible materials, but rather to present some representative and advantageous examples. More details can be found at various review articles focusing on this field [105].

**Figure 5:**
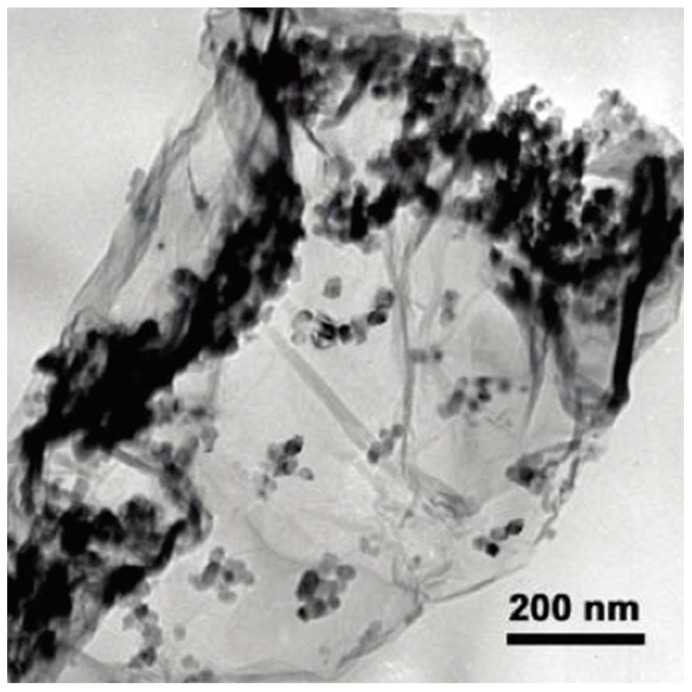
Composite of TiO_2_ P25 nanoparticles decorated on reduced graphene oxide following a hydrothermal process which showed an elevated photodecomposition performance against methylene blue both under ultraviolent and visible light irradiation. Reprinted with permission from Ref. [106]. Copyrights Elsevier, 2009.

Among the most broadly studied combination is with titanium dioxide. In the innovative work by Zhang et al. published in 2009, commercially available TiO_2_ nanoparticles (P25) were chemically bonded on graphene oxide following an one-step hydrothermal process [106]. The successful decoration (predominately concentrated along-side the wrinkles) of the TiO_2_ nanoparticles on single layers of reduced graphene oxide as can be seen at the TEM image in Figure 5. The composite showed significantly higher adsorptive as well as photocatalytic removal efficiency against methylene blue (MB) azo dye compared to bare P25 and a composite of P25 with carbon nanotubes both under ultraviolent and visible light irradiation. Szabó and co-workers synthesized composite of TiO_2_ P25 with exfoliated GO by heterocoagulation [107]. Even though the incorporation of GO did not have a positive impact on the photo-oxidation of phenol, the authors presented that the composite showed faster sedimentation of the composite, a property that is useful when utilizing nanoparticles of the size of P25 (20–70 nm) in real (waste)water treatment, since the separation of the catalyst can be achieved easily avoiding the formation of colloidal suspensions.

Minella et al. synthesized also TiO_2_-(r)GO composite (TiGOcomp) by chemical reduction of a GO/TiO_2_ dispersion by hydrazine [108], where the addition of graphene-phase had a negative impact on phenol photo-removal, but on the contrary a positive on methylene blue photo-elimination both under UV or Vis light exposure. The authors concluded that the adsorption phenomena play a key role, since for instance the photoactivity of the composite under visible light only in the case of the dye was due to its adsorption on the surface initiate a dye-sensitized mechanism of light absorption. Upon the synthesis of the TiO_2_-(r)GO composites, various factors matter towards elevation of photoreactivity. Except for the optimization of the graphene-phase in wt.% of the final material, the thickness of the graphene part is important. Aleksandrzak and co-workers showed that the optimum photo-oxidation performance against phenol under visible light was obtained when the photocatalyst consisted of single reduced graphite oxide layers while, as the graphene phase was became more bulky, the photocatalytic activity was diminishing [109]. This trend was assigned to a better dispersion of the inorganic phase on the rGO sheets leading to more available catalytic sites as well as positively affecting the separation of the photoinduced electron/hole pairs due to their higher mobility between the two phases.

**Figure 6:**
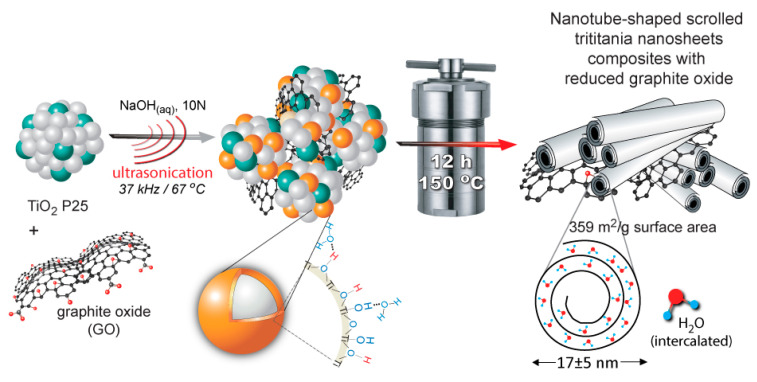
A schematic illustration presenting the followed steps for the synthesis of composites of titanate nanotubes (scrolled titanate nanosheets in nanotubular shapes) with reduced graphite oxide as filler (TiO-NTbs@rGO). Reprinted with permission from Ref. [111]. Copyrights Elsevier, 2021.

Utilizing TiO_2_-(r)GO composites was also shown as a prosperous strategy for environmental remediation application in gaseous phase. TiO_2_ P25 composites with reduced graphite oxide (rGO) were prepared following a single one-pot ultrasound (37 kHz) treatment process in basic solution [110]. The ultrasound treatment resulted in the reduction of GO, creation of an amorphous phase at the outer surface of the TiO_2_ nanoparticles rich in acidic and basic hydroxyl groups, while Ti-O-C bonds were formed between TiO_2_ nanoparticles and the graphite phase. The as referred P25GO-US composites showed a higher by 67% photocatalytic ability against toxic vapors of 2-chloroethyl ethyl sulfide, a surrogate of the Chemical Warfare Agent Mustard gas, compared to pristine P25, while P25GO-US noteworthily outperformed other advantageous materials like barium titanate nanospheres, zinc peroxide nanoparticles, or even composite of zirconium-based Metal-Organic Frameworks and rGO, examined under the same conditions. Going a step ahead, further hydrothermal treatment of P25GO-US at 150 °C led to the transformation of the TiO_2_ nanoparticles to titanate nanosheets (H2Ti_3_O_7_), which were self-scrolled forming titanate nanotubes composites with rGO (TiO-NTbs@rGO), as can be seen in Figure 6 [111]. The synthesized nanocomposite with the optimum amount of rGO (4 wt%) showed elevated specific surface area of 359 m^2^/g, which was higher by 23% compared to pure titanate nanotube. The photocatalytic ability and even more importantly the photo-initiated oxidation reactivity were improved by the presence of the rGO phase since various compounds were formed as a result of the radicals’ formation. Titanate nanotubes composite with GO were also studied in aqueous applications. For example, Anirudhan et al. prepared a nanocomposite of titanate nanotubes silylated GO molecularly imprinted polymer that presented a high photocatalytic activity against a commercial herbicide, 2,4-Dichlorophenoxyacetic acid under visible light and even up to five cycles of experiments [112].

**Figure 7:**
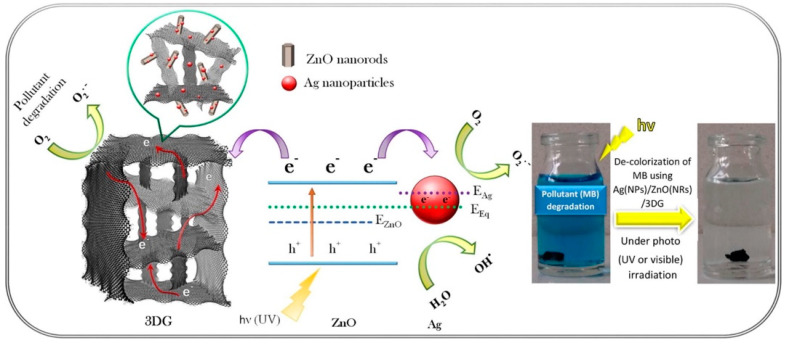
Mechanism of action of photocatalytic degradation of methylene blue dye by Ag/ZnO/3DG graphene structure. Reprinted with permission from Ref. [114]. Copyright 2019, Elsevier.

Beside TiOx-based materials, graphene phases were used for designing and preparation of composite with other metal oxides. For instance, Mei et al. [113] reported a one-pot hydrothermal protocol followed with a freeze-drying method in order to synthesize a ZnO hybrid with 3D graphene aerogel (GA). This hybrid material showed high photochemical stability and significantly higher photodecomposition ability against MB dye (under visible or ultraviolent light irradiation) compared to pristine ZnO as well as compared to a plethora of zinc oxide-based materials. To further improve the photo-reactivity, Kheirabadi et al. also incorporated Ag nanoparticles (of size ~60 nm) in a composite build on zinc oxide nanorods and three-dimensional graphene network (3DG) following a combined hydrothermal-photodeposition procedure [114]. The final mixed composite, Ag/ZnO/3DG (Figure 7), showed significantly higher photodecomposition capability against MB both under visible and UV light irradiation. The authors pointed out three important effects arisen from the presence of the 3D graphene network. Firstly, the retardation in photoinduced species recombination due to the electronic conductivity of 3DG. Secondly, the π-π interactions of the dye molecules with the 3DG due to their aromatic structure. Lastly, the easiness of the separation and recovery of the material after the experiments. The elevation of the photoreactivity was also linked to the extended formation of superoxide and hydroxyl radicals. It should always be considered that the addition of graphene towards composite formation results in a higher dispersion and hence availability of the active catalytic sites as well as in an increase of the specific surface area of the final material compared to the pure counterparts [115,116,117].

Various other novel and advantageous composites based on different inorganic phases and graphene derivatives were also studied. Cai et al. [118] reported a Cu_2_O/rGO composite aerogel, which showed a more than two folds higher photocatalytic degradation of methyl orange (MO) dye under visible light irradiation as compared to Cu_2_O nanoparticle. In another work, nickel-loaded TiO_2_ nanoparticles were decorated on graphene oxide by a two steps microwave-based process [119]. Optimization of the Ni loading showed the best one to be 50 wt%, with the new catalytic NiTiO_3_ sites to cause uplifted adsorptive and photocatalytic performance against Rhodamine B under visible or UV exposure. Plenty other metal oxide/graphene-derivatives composites used for (waste)water treatment applications can be found elsewhere [52,120].

Only a few years ago, Petit and Bandosz presented a series of different composites consisting of Metal-Organic Frameworks (MOFs) and graphite oxide utilized initially for air purification applications [121], since one crucial drawback of various MOFs is their instability in aqueous matrixes [122,123]. Although, in the last decade, the materials design strategy to utilize water stable MOFs in order to synthesize composite with graphene derivatives for various applications, especially in catalysis, showed an incremental trend of interest. Huang and Liu followed a simple solvothermal method to synthesize a hybrid material consisting of (reduced) graphene oxide and Ti-based MOF, specifically NH_2_-MIL-125(Ti) [124]. The obtained material (rGO–NMTi) showed significantly higher photocatalytic removal efficiency against MB under visible light comparing to the counterparts separately, due to the arisen synergistic effects. A composite of Zn-based MOF (MOF-5) and rGO, obtained by a one-step hydrothermal method, also showed much higher photodegradation performance than that of the components separately, against various dyes (MB, PhB, and MO) under solar irradiation [125]. The anchored on the MOF surface rGO sheets led to a droppage by 60% of the photoluminescence efficiency of the composite versus the pure MOF due to the retardation of the e-/h+ pairs recombination. In another work, a MIL-68(In)-NH_2_/GO composite was synthesized by a solvothermal method and its photodegradation efficiency against antibiotic amoxicillin (AMX) was studied [126]. It was pointed out that graphite phase can have an alternative role towards elevation of the photocatalytic activity by acting as sensitizer to improve the visible light absorptivity.

More complex composites were also showed to have a superior photoreactivity against organic compounds. For instance, nanoparticles of silver ferrite (AgFeO_2_) were in situ impregnated onto CuBTC/HKUST-1 MOF phase and/or graphene to obtain binary or tertiary heterojunction catalysts [127]. The tertiary photocatalyst (AgFeO_2_/Gr/CuBTC) outperformed the binary counterparts when tested for amoxicillin (AMC) or diclofenac (DCF) sunlight-assisted removal. The increase of the photoreactivity was attributed to the charge carriers transfer from CuBTC conduction band to AgFeO_2_ valence band with graphene phase to promote the charges delocalization (via a Z-scheme mechanism) by acting as electron acceptor/mediator, leading also to a smaller in value band gap without altering the morphological and crystalline structure. A mixed Bi_2_O_3_/CuBTC/GO displayed also a great photocatalytic activity against RhB under visible light, since the photoinduced electrons were transformed via the graphene phase from the Bi_2_O_3_ phase to the MOF phase where the adsorbed organic molecules were decomposed [128]. Analogues results were obtained when a different combination was used to form a ternary photocatalyst, namely GO, BiVO_4_ as the semiconductor phase, and MIL-53 (Fe) as the MOF phase [129]. Both semiconductor/MOFs/carbonaceous-based materials showed good stability and reusability.

Graphite derivatives, solely or as counterparts in composites, were also presented as prosperous candidates as catalyst for ultrasound (US) assisted removal of organics from (waste)water matrixes. Yeomin Yoon and co-workers showed that the presence of GO has a positive impact on the sonocatalytic removal of diclofenac (DCF) and carbamazepine (CBZ) under three different US frequencies (28, 580, and 1000 kHz) [103]. Sonication affected positively the chemical decomposition (sonodegradation) of the organics by the formation of active radical species like hydroxyl radicals. Additionally, an indirect effect was the extended dispersion/exfoliation and hence elevation of the adsorption sites number. Ultrasound irradiation at 28 kHz showed the best results on the regards of adsorptive removal, while 580 kHz led to the highest sonodegradation due to radicals’ formation. Babu et al. [130] studied the sonophotocatalytic degradation of MO in the presence of CuO-TiO2/rGO under UV (312 nm and 365 nm) or visible light and ultrasonic irradiation of 40 kHz. The results revealed that the assistance of ultrasound power significantly improved the photocatalytic degradation of MO. The coupling of sonolysis and photocatalysis revealed a synergistic effect, since the sonophotocatalysis efficiency was drastically higher than that of photocatalysis by 3.7 folds. Khairy et al. [131] examined the photocatalytic, sonocatalytic and sonophotocatalytic activity of ZnO/GO composite for the degradation of 4-nitrophenol under visible light illumination and ultrasonic radiation (20 kHz). Ultrasonication was found to increase the decomposition rate, since the highest rate was found in the case of the sonophotocatalysis, which was almost three folds higher than simple photocatalysis.

A magnetically separable ternary composite consisting of copper ferrite (CuFe_2_O_4_) nanoparticles, MIL-101(Cr) MOF and GO, synthesized via a two-steps hydrothermal process (at 200 °C), revealed an elevated sonocatalytic reactivity for decolorization of water samples with high concentration of dyes like rhodamine B, methyl orange, and methylene blue, using H_2_O_2_ [132]. More specifically, the nanocomposite increased sonocatalytic ability, which was linked mechanistically to hot spots formation and sonoluminescence effects leading to enhancement on the hydroxyl radicals (•OH) formation closed to the materials surface which decompose the adsorbed dye-molecules. A crucial and practical property for potentially utilization in large-scale industrial scale is the easiness of separation from the dispersion due to the magnetic nature of the nanocomposite.

The usage of bimetallic nanoparticles of Au and Ru supported on rGO was proved to exhibit a more “green”-oriented photocatalytic efficiency towards the formation of 2,5-diformylfuran either from partial oxidation of HMF or from a one-pot base-free conversion of fructose even under natural sunlight [133]. The high photoreactivity of the nanocomposite was linked to the transportation of the photo-excited electrons from Ru to Au nanoparticles via the conductive graphene phase since the monometallic composites of Ru or Au with rGO demonstrated significantly lower photoreactivity. The optimum ratio of Ru to Au was found to be 5:1.

### 3.4. Graphitic Carbon Nitride (g-C_3_N_4_) Based Nanocatalysts

Within the active goals of research and development in catalysis is to decrease the cost and the environmental footprint of the materials, and if possible, to design efficient metal free catalysts. It was the inspirational work by Markus Antonietti and co-workers in 2009 who demonstrated the metal-free polymeric graphitic carbon nitride (g-C_3_N_4_) as an efficient photocatalyst for hydrogen production under visible light exposure using a sacrificial donor [134]. Afterwards, chemically modified forms of g-C_3_N_4_, solely or as hybrid and/or composite materials, were studied broadly and intensively for a plethora of photocatalytic applications, including photocatalytic environmental remediation and biomass valorization [135,136,137,138]. In general, g-C_3_N_4_ is a semiconductor material, consists of carbon and nitrogen, and has a high chemical and thermal stability due to the strong covalent linkage between carbon and nitrogen atoms. It has a moderate bandgap energy of around 2.7 eV, and hence possesses the ability to absorb light in visible range [139]. g-C_3_N_4_ has a layered structure like graphene buildup predominately from s-triazine (C_3_N_3_ ring) and tri-s-triazine (heptazine ring: C_6_N_7_) units, known also as melem. Various N-containing functional groups existing at the edge or as defects (such as >N–N<, =C–N<, =N–, –NH– and –NH_2_, etc.) and can act as active sites for adsorption, catalysis or either as bridges for bonding with various other phases for the formation of composites [137,140,141,142,143]. The physicochemical features can be tuned by various approaches as for instance by changing the precursors, the thermal polymerization protocol of synthesis, or by post-synthesis methods. There are numerous articles regarding utilization of g-C_3_N_4_ for the formation of composites with inorganic phases and their application in water treatment in order to photochemically remove organic pollutants, such as pharmaceuticals (oxytetracycline, diclofenac), dyes (rhodamine B, methylene blue, methyl orange), aromatics (phenol, bisphenol A) and more. Some of the latest reported cases are collected in Table 2.

Among the ultimately important goals towards designing of novel and selective green-nanophotocatalysts, is to engineer precisely the structural, morphological, and surface chemistry features of g-C_3_N_4_ by introducing heteroatoms, but not metals, as well as by creating defects and nanostructured morphological and structural features. For example, Zhang et al. [144] synthesized a functionalized g-C_3_N_4_ by doping with single non-metallic element B or P, or by co-doping with both B and P. The latter material showed significantly higher photodecomposition activity against oxytetracycline (OTC) and Rh-B comparing to single-doped and un-doped counterparts. The main reasons were the narrowing of the band gap to 2.61 eV and the morphology of co-doped composite that improved the transfer and separation of photogenerated electron-hole pairs. Co-doped with S and O mesoporous-structured crimped nanosheets of g-C_3_N_4_ obtained by polymerization of melamine using hydrogen peroxide bonded thithiocyanuric acid showed a higher by six folds visible light driven photodecomposition of RhB compared to pristine g-C_3_N_4_ [145]. The authors presented that the increased photoreactivity was due to the delocalization of the LUMO and HOMO and narrowing of the bandgap resulted from the S and O doping. In another inspiring work, Pawar et al. used a template-free chemical (at room temperature) method for the preparation of nano-porous one-dimensional microrods of g-C_3_N_4_ [146]. The role of acids was to induce etching and delamination, as well as to create the nanopores via oxidation and protonation. In comparison to the non-modified counterpart, the nanoporous microrods revealed five folds increased photocatalytic decomposition efficiency against MB dye under visible light exposure and an ~26 times greater photocatalytic H_2_ production rate, reaching 34 μmol/g. The elevated photoreactivity was attributed predominately to the prolonged lifetime of the photoinduced charge carriers and to the increment of the active catalytic-sites number, which were interconnected due to the formation of the nanopores.

Oxidation of bulky g-C_3_N_4_ following the most well-known method for the synthesis of graphite oxide from graphite, namely Hummers method, led to the formation of nanospheres, 5 to 50 nm in size, consisting of graphitic carbon nitride layers reach in oxygen containing functional groups at the edges like nitroso, sulfonic, hydroxyl, and carboxyl [141]. The obtained oxidized graphitic carbon nitride nanosphere, referred to as gCNox, showed more than double adsorption efficiency and rate of photocatalytic decomposition of toxic vapors of a surrogate of mustard gas. Going a step onward, gCNox was used as a composite feeler to synthesize novel composites based on MOF, as for instance HKUST-1 or UiO-66 phases [116,117,142] or with other inorganic nanostructures such as zinc peroxide nanoparticles (ZnO_2_/gCNox) [147]. In the case of the MOFs, their composites showed alteration of the physicochemical features important for photocatalytic applications such as improved porosity and surface chemistry heterogeneity. Especially in the case of HKUST-1/gCNox nanocomposites, their decoration on cotton textiles led to “smart” multifunctional textiles capable to simultaneously adsorb, decompose, and sense/detect toxic vapors of low concentration [148]. The ZnO_2_/gCNox composite showed the unique capability of day-night photocatalysis, known also as dark photocatalysis, where the photoexcited electron formed at the gCNox phase are transferred and stored in the inorganic phase, which can be reactive even in the dark [147].

The photocatalytic selective partial oxidation of HMF to DFF in aqueous medium using g-C_3_N_4_ was firstly reported in 2016 by Krivtsov et al. [149]. It was revealed by studying different precursors for the preparation of g-C_3_N_4_ such as melamine, urea, and thiourea, that the melamine derived material yielded the highest amount of DFF (10.9% yield), with a 30% of DFF selectivity under artificial light exposure of a range 340–420 nm (emission peak at 365 nm). Thermal exfoliation had a positive impact and the obtained porous materials after treatment at 540 °C (MCN-540) showed the highest HMF conversion (69%), DFF selectivity (49%) and hence DFF yield (39.7%). Upon real outdoor light exposure, MCN-540 reached >99% HMF conversion and 49% selectivity. Using p-benzoquinone as O_2_^•–^ - scavenger, it was found that O_2_^•–^ were the main reactive species involved in the HMF to DFF selective photo-oxidation.

A very short period afterwards, Wu et al. also studied the selective HMF to DFF conversion by g-C_3_N_4_ obtained from melamine after calcination at 540 °C and going a step forward, they post-synthetically treated with water and further 500 °C calcination (g-C_3_N_4_-w) in order to elevate the porosity [150]. The as received g-C_3_N_4_ showed under visible irradiation (>400 nm) a 29.9% HMF conversion and 21.1% DFF selectivity (6.3% DFF yield) in acetonitrile and benzotrifluoride (PhCF_3_) mixture as solvent with continuous O_2_ purging (10 mL/in). Even though g-C_3_N_4_-w showed similar HMF conversion (31.2%) under the same conditions, the DFF selectivity and the yield were dramatically higher, 85.6 and 26.7%, respectively. Additional light irradiation in ultraviolent region (360–400 nm) had a positive impact on the HMF conversion (85.6%) and a negative one in DFF selectivity (47.2%). When water was used as solvent, the HMF conversion did not altered significantly, but the DFF selectivity was minished to around 28%, and hence the yield was only ~8%. In this work, it was also pointed out that the O_2_^•–^ species were predominately acting as oxidation agents, while purging with N_2_ led to blockage of the reaction (2.3% HMF conversion).

Ilkaeva et al. prepared hydrogen peroxide modified g-C_3_N_4_ by thermal etching melamine derived graphitic carbon nitride to increase the specific surface area, then chemically treating with H_2_O_2_ and finally by thermal treatment at different temperatures (200, 300, and 400 °C) [151]. Even though the peroxide-adduct materials showed a lower photocatalytic HMF conversion either under UV light (365 nm) or natural solar light (SL), the selectivity and hence the yield were noticeably enhanced. The best performing sample was the one received after thermal treatment at 300 °C, which presented 20 and 51% HMF conversion under UV and SL, respectively, while the DFF yields were 15 and 38%. The untreated with H_2_O_2_ material revealed 18 and 15% DFF yield, respectively, under UV or SL. Last but not least, it should be mentioned that g-C_3_N_4_ was used as platform/substate for the (photo)deposition of Pt nanoclusters (~3% wt.%) with the materials to presented great solar-driven HMF to DFF conversion with simultaneous H_2_ production by water splitting [152,153]. Interestingly, the material showed negligible HMF conversion in water when then system was degassed under vacuum [153].

Graphitic carbon nitrite was used as composite filler or/and as substrate for the synthesis of composites, which showed photocatalytic activity towards HMF to DFF selective oxidation. Vanadium, iron, Nb_2_O_5_, NaNbO_3_, WO_3_, and BiWO_3_ doping was shown to lead to active materials and, especially in the case of the vanadium doping, DFF was obtained in a good yield starting from fructose [154,155,156,157,158,159]. It was presented lately that composites of g-C_3_N_4_ with MXenes can achieve elevated DFF from HMF formation as well as oxidation of other aromatic or non-aromatic alcohols, such as oxidation of p-methoxybenzyl alcohol to p-methoxybenzaldehyde (96% selectivity) [160].

### 3.5. Other Carbon-Based Materials

On the regards of environmental remediation applications, other kind of carbonaceous materials, especially based on porous carbon or/and biochar, was shown to have ultimately high adsorptive capability against not only organic pollutants, but also against (heavy) metals [161,162]. The recent trend on materials design is to utilize undesired biomass and wastes as feedstock and after thermal treatment (carbonization) or/and activation to obtain bulky carbons/biochars [20,163,164,165] with the ultimate goal of the final material to possess high surface chemistry heterogeneity, high porosity and ultimately a micro-/nano-porous nature [12,13]. However, this aspect is outside the scope of this work, since the literature is very abundant in this kind of high value reviews and research articles. At this sub-chapter, we present some representative examples other than the ones mentioned in the previous paragraphs of carbonaceous materials with emphasis on their utilization as substates or composites/hybrid fillers for photocatalytic or sonocatalytic applications, as well as in (photo)catalytic HMF oxidative valorization.

Perciani de Moraes et al. studied the synthesis of composites in which Bi-doped ZnO/β-Bi_2_O_3_ phases were incorporated on carbon xerogel under a wide range of carbonization temperature (300–600 °C) [166]. The carbonization temperature decisively influences the physicochemical features and the photocatalytic ability of the final material under solar and visible light irradiation even though the surface area of the obtained composites was below 30 m^2^/g for all samples. The best performing sample for the photodecomposition of 4-chlorophenol was found to be the one obtained after carbonization at 600 °C, due to the extended formation of β-Bi_2_O_3_ and Bi^0^ phases. Their presence led to the formation of heterojunctions between the inorganic and organic phase of the material, favoring the charge mobility. The optimum amount of Bi was around 5 wt.% and the formation of hydroxyl radicals played a predominant role on 4-chlorophenol photodecomposition as determined by scavengers’ tests. Wang et al. [167] synthesized a ZnO/carbon nanocomposite with 3D hierarchical micro-, meso-, macro-porous structure following a dual templating method (combination of ice and micelle-templation) and at the end pyrolysis. They studied the photocatalytic degradation of MB dye under ultraviolent and visible light irradiation and the results exhibited that the nanocomposite showed a higher photocatalytic activity under both UV and visible light irradiation, which was 2.2 and 7 folds higher compared than that of a control ZnO with carbon mixture. Ultrasound irradiation (50 kHz) had a positive impact on the photocatalytic MB degradation efficiency mainly due to formation of more OH• radicals.

For the ultrasonic-assisted degradation of pharmaceutical products such as naproxen (NPX) and acetaminophen (AAP), Im et al. [168] used biochar (BC) and powdered activated carbon (AC). Among the three frequencies of 28, 580, and 1000 kHz, the highest catalytic degradation was achieved at 580 kHz, with BC possessing the higher degradation efficiency against both pharmaceuticals as compared to powdered AC. Density function theory and molecular modeling analysis calculations revealed that NPX degradation has faster kinetics comparing to AAP. When the BC or AC was exposed to the ultrasonic irradiation, the materials’ surfaces act as active site where the formed OH• react with the adsorbed organic molecules. The crucial effect of hydroxyl radicals was verified by the addition of tert-butanol, which by acting as hydroxyl radicals’ scavenger led to a diminishing of the pharmaceuticals’ degradation extend, while the degradation blockage was significantly limited using methanol as scavenger. Khataee et al. [75] synthesized composites consisting of TiO_2_ and biochar (TiO_2_-BC) and study them as sonocatalysts at 40 kHz. When RB69 solution was treated by ultrasonication, the sono-degradation extend was 11%. Addition of BC and TiO_2_/BC promoted RB69 decomposition, reaching 63 and 98%, respectively. The authors explained the sono-decomposition increase due to hot spots formation and sonoluminescence phenomena that affect positively the generation of free OH• radicals, which were determined using inorganic salts (NaCl, Na_2_SO_4_ and Na_2_CO_3_) as scavengers. More precisely, they revealed by monitoring all the formed products during the catalytic experiments that RB69 molecules get oxidized initially to aromatic compounds that are transformed to aliphatic and finally mineralized to CO_2_ and H_2_O. Lisowski and co-workers synthesized using ultrasound irradiation an organic–inorganic hybrids consisting of TiO_2_ and biochar, with the latter derived from wood and straw pellets [169]. The composite showed elevated phenol degradation both under ultraviolet and visible light irradiation. Moreover, the composite revealed high photocatalytic activity for the selective oxidation of methanol gas flow to methyl formate.

Singh et al. synthesized TiO_2_/activated carbon nanocomposites and studied their sonocatalytic, photocatalytic and sonophotocatalytic degradation efficiency against the Direct Blue-199 (DB-199) dye by US irradiation of a frequency of 26 kHz and UV irradiation (8 W) [170]. The best performing synthesized nanocomposite showed a 98% sonophotocatalytic removal, while the sonocatalytic and photocatalytic removal efficiencies were 94 and 92%, respectively. It should pointed out that except the direct arisen chemical and physical effects of ultrasonication, it should be considered that indirect effects (as for instance temperature increase) or aspects hard to be monitored (elevated de-aggregation of the catalyst’s particles) should be considered, as it was showed previously [61].

In 2011, Davis et al. [171] studied the catalytic efficiency of noble metals (Pd, Pt, and Au) supported AC for the successful catalytic HMF oxidation. Although, the addition of NaOH in order to achieve basic environment and high pressure of O_2_ (690 kPa) were necessary. Gold deposition on AC of ~3 wt.% led to the highest oxidation extend and rate of the HMF’s aldehyde group to carboxylic, although the hydroxyl group was not oxidized. Pt or Pd addition showed to promote the oxidation of the hydroxyl group leading to the full oxidized product FDCA. In order to be aligned with the modern trends of “green” and sustainable and avoid the use of noble metals and high pressures, Liu and coworkers synthesized a mesoporous carbon decorated with well-distributed CoO_x_ nanoparticles of around 25 nm diameter [172]. The synthesis involved the ion-exchange of Co^2+^ from [Co(NH_3_)6]^2+^ with H+ from a polymeric gel and the final calcination of the received Co-doped polymeric framework. The as-synthesized final composite nanomaterial revealed a superior efficiency for the oxidation of HMF to FDCA in water as solvent and O_2_ as the oxidizing agent, with the HMF conversion to reach 98.3% and the FDCA yield 95.3% after 30 h of reaction at 80 °C. This work was a big step towards environmentally friendly and cost-effective catalytic approached for the HMF oxidation in aqueous environment using mesoporous carbons as substate for the fabrication of highly active materials for furfural oxidation.

Going a step forward and in order to take the advantage of photochemistry, TiO_2_ nanoparticles were embedded in chemically sulfur functionalized porous carbon as support [173]. After 8 h of reaction at 70 °C under molecular oxygen purging (1 atm), a 91% HMF conversion was achieved with the DFF selectivity to be 88%. Further extension of the reaction’s duration to 22 h at 85 °C led to absolute HMF conversion, with the selectivities to DFF, FDCA, and FFCA (5-Formyl-2-furancarboxylic acid) to be 13, 23, and 64%, respectively. The use of sulfonated carbon, the presence of acid sites and the enhanced availability of titania with +4 oxidation state found to play a key role for the elevated and selective HMF conversion.

Another category of carbon allotrope is fullerenes, which are predominantly spherical or ellipsoidal nanostructures. The most well-known representative of this family is the buckminsterfullerene C_60_ and it is used for the preparation of novel nanocomposite catalysts, especially combined with semiconductor phases [174]. C_60_ possess a conjugated structure with high density of delocalized electrons, and hence high electron mobility (>1.3 cm^2^V^−1^S^−1^) [175]. Bai et al. [176] synthesized a composite consisting of C_60_ and modified graphitic carbon nitride (g-C_3_N_4_), and they studied its photocatalytic activity for degradation of MB dye and phenol under visible light illumination. The results of photocatalytic activity exhibited that the C_60_/g-C_3_N_4_ nanocomposite showed higher degradation efficiency for both organics comparing to the bulky g-C_3_N_4_. This enhancement was linked to the increased rate of photogenerated electrons transfer and charge separation ability of C_60_ in the composite due to the formation of heterojunctions. Another result upon composite formation is that the valance band of the g-C_3_N_4_ was shifted to lower energy in the composite, which also affect positively the photocatalytic activity and so the formation of active radicals. Combination of fullerene with inorganic nanophases was also shown as a successful strategy towards enhancement of the photocatalytic capability as a result of composite formation. For instance, Li et al. [177] reported the synthesis of Bi_2_TiO_4_F_2_ nanoparticles and a composite with fullerene (C_60_), and they studied the photocatalytic degradation of Rh-B and Eosin Y (EY) under visible light illumination. The results exhibited that the C_60_/Bi_2_TiO_4_F_2_ composite nanocatalyst showed a significantly higher photocatalytic activity than the Bi_2_TiO_4_F_2_. The higher photocatalytic degradation was attributed to the formation of strong heterojunction between Bi_2_TiO_4_F_2_ and C_60_, which leads to the inhibition of photogenerated electron–hole pair recombination and enhance light utilization. An overview of different CnCs utilized for the photocatalytic oxidation of HMF is presented in Table 2.

**Table 2:** Photocatalytic oxidation of HMF using novel CnCs.

Catalyst	Solvent	Experimental				HMF Conversion (%)	DFF Selectivity (%)	Ref.
		Catalytic Loading (g/L)	Time (min)	Light Source	Concentration			
Bi_2_WO_6_/mpg–C_3_N_4_	water	10	8	Vis.	0.1 mM	59	84	[158]
g-C_3_N_4_/NaNbO_3_	water	10	8	Vis.	0.1 mmol	35	87	[157]
mesoporous carbon nitride	water	-	48	Vis.	0.1 mmol	38	99	[152]
ultrathin graphitic carbon nitride	water	1	5	Vis.	10 mM	48	95	[153]
Ni/CdS	water	1	22	Vis.	10 mM	22	100	[178]
Au-Ru nanoparticles decorated reduced graphene oxides	toluene	4	8	Vis.	0.5 mmol	95.7	95	[133]
WO_3_/g-C_3_N_4_	ACN (3 mL) + PhCF3 (2 mL)	10	6	Vis.	0.1 mmol	27.4	87	[156]
MXene/g-C_3_N_4_ composite (MX/CN)	benzotrifluoride	10	10	Vis.	5 mM	32	90	[160]

## 4. Lignin Hydrogenolysis to Valuable Phenolic Compounds

Lignin is an amorphous phenolic polymer and one of the three major components of the lignocellulosic biomass. The macromolecule of lignin is formed by the polymerization of three methoxylated monolignols: the coniferyl, the sinapyl and the p-coumaryl alcohol via the phenylpropanoid pathway [179]. The three lignin monomers are linked via ether (β-O-4, α-O-4, 4-O-5) and carbon–carbon (β-β, β-5, β-1) bonds. The lignin content, the inter-unit linkages type and abundance as well as the monolignols types are strongly depended on the biomass type. Softwood type biomass exhibits high lignin content (27–33%) with coniferyl alcohol as the main building block, linked mainly with carbon–carbon bonds [180]. On the contrary, hardwood type biomass exhibits lower lignin content (18–25%) with both coniferyl and sinapyl alcohols as building blocks, linked mainly with β-O-4 bonds [180]. Lignin isolation process and properties can determine the potential valorization towards value added chemicals via thermo or bio-catalytic processes. Considering the thermochemical processes, lignin conversion can be achieved via fast pyrolysis, liquid phase depolymerization under oxidative/reductive conditions and hydrotreatment in the absence of solvent [179,181,182,183,184,185,186].

Reductive depolymerization of lignin involves the hydrogenolysis of C–O and C–C interunit linkages, which is usually occurred in the presence of a catalyst under hydrogen pressure [187]. The main product of the reaction is a bio-oil rich in substituted guaicyl and syringyl type compounds as well as alkylated phenols [182]. Process parameters, e.g., reaction temperature and time, solvent, hydrogen source and pressure, can significantly influence the conversion and the monomers yield. Catalyst, also plays important role in the depolymerization degree and the bio-oil composition. A variety of catalysts based on precious (Pd, Pt, Ru, Rh) and transition metal (Ni, Mo, Cu, Fe, Co) have been widely used in lignin depolymerization. Noble metal catalysts proved to be very active catalysts for lignin depolymerization reactions, but the high cost and the deep hydrogenation ability, led to their replacement by transition metals [188]. Well-defined molecular catalysts were first used for the selective cleavage of lignin-derived aryl ether linkages. Despite their facility to react with individual ether linkages, the difficulty of their separation/removal from liquid products increases the cost and complexity during biomass conversion [189]. Hence, an extensive range of heterogeneous catalysts, including carbons, zeolites, as well as Al and Si oxides, have been developed to achieve the selective cleavage of ether linkages in lignin. These catalysts can be used either as their own or as metal supports.

### 4.1. Activated Carbons

Metal catalysts supported on activated carbons (ACs) are promising catalysts leading to (i) less coking; (ii) lower cost; (iii) possibility of recovering the active metals from catalysts by burning off the support, and (iv) the possibility of being produced from lignocellulosic biomass [190]. ACs are usually neutral materials with micro/mesoporous properties that have been used as supports for both transition and noble metal catalysts and proved to have beneficial effect on the depolymerization of lignin (Table 3). Sanyoto et al., examined the effect of SiO_2_, zeolite ZSM-5, AC, carbon aerogel, ZrO_2_, and Al_2_O_3_ as supports for platinum [191]. Pt/AC exhibited the highest monomer yield (27.6%) compared to the other supports which resulted in significantly lower monomers yield in the range of 3.4–19.5%. The superior activity of Pt/AC was attributed to the large Pt surface area (90.5 m^2^/g), measured by CO-chemisorption. In a similar research, AC was proved to enhance the accessibility to the metallic sites compared to Al_2_O_3_, thus increasing the bio-oil yield from 28.0 wt.% (5%Ru/Al_2_O_3_) to 31 wt.% (5%Ru/AC) [192]. Comparing the activity of Ru and Pd/AC in the depolymerization of acid hydrolysis lignin, 5%Ru/AC was proven to be a more active catalyst and resulted in higher bio-oil yield (31 wt.%) than 5%Pd/AC (19.1 wt.%) [192]. The higher activity of Ru is attributed to its higher hydrogenolysis/hydrogenation ability.

Noble metals supported on activated carbons exhibit high activity in the reductive depolymerization of lignins. Bare Pd/AC resulted in 6.8% monomers yield from alkali lignin, while the catalytic activity can be enhanced with the addition of metal chlorides (MCl_x_), which act as acid centers and are responsible for the breakage of the low activity energy chemical bonds in lignin molecule. Pd/C combined with CrCl_3_ exhibited the highest monomer yield (28.5 wt.%) during alkali lignin hydrogenolysis [193]. Similar results are reported for hydrolysis lignin depolymerization, where the activity of 5%Pd/AC was enhanced with CrCl_3_ resulted in 26.3 wt.% monomers yield [194]. In presence of Pd/C, corn stover lignin can be converted into phenolic monomers with 30 wt.% yield [195]. Lower yields, in the range of 18.0–20.1 wt.%, are obtained over Pd, Pt and Ru supported on activated carbons, in the organosolv lignin hydrogenolysis at 200 °C [196]. Slightly lower activity was obtained by the bimetallic catalysts Ni-Fe/AC, with monomers yield in the range of 17.7–20.3 wt.%, higher than the monometallic Ni and Fe/AC, with monomer yields 12.54 and 6.3 wt.%, respectively. Considering the bio-oil composition, dihydroxylation reaction seems to be enhanced in the presence of Ni-Fe/AC [196].

Monometallic 5%Ni/AC exhibited low monomer yield (12.1 wt.%) in the hydrogenolysis of corncob lignin under the optimized reaction conditions while the main products were alkoxy phenols, including propyl/propenyl guaiacol and syringol (2.7 wt.%), mono-phenols of ethyl/vinyl phenol and guaiacol (4.5 wt.%), methyl coumarate/ferulate as well as unsaturated phenols (31%) [197]. Enhancement of nickel activity in the hydrogenolysis of organosolv poplar lignin has been also achieved by copper addition. The highest bio-oil yield (77.2 wt.%) and the minimum amount of char were achieved over 10 wt.% Ni-5 wt.% Cu/AC bimetallic catalyst, where the main reaction products were alkylated oxyphenols [198]. The catalyst exhibited better hydrogenation activity, inhibited repolymerization reactions and sufficient stability to be reused for at least three successive runs. Both monometallic and bimetallic catalysts exhibited mesoporous properties which facilitate the mass transfer of the reactants. However, tungsten addition did not improve Ni/AC activity, which resulted in lower bio-oil yield (61.9 wt.%), while none phenolic monomers were detected [188]. Probably, tungsten nanoparticles promoted the adsorption of reaction solvents (EtOH and IPA), blocking the access of lignin to the catalyst. The synergistic effect of Ni-Cu/AC in the hydrogenolysis of organosolv lignin was also highlighted by Zhang et al. Under the optimized conditions, 5% Ni-5%Cu/AC resulted in maximum bio-oil yield (40.2 wt.%) while the activity was attributed to the interactions between the two metals and the support. More specifically, Cu is susceptible to electrons provided by Ni, thus improving the dispersion of Cu nanoparticles and their number on the catalyst surface. Simultaneously, Cu can change the crystal structure of Ni and effectively reduce the excessive hydrogenation products [199].

**Table 3:** Lignin hydrogenolysis over metallic catalysts supported on activated carbons.

Catalyst	Lignin	Reaction Conditions			Monomer Yield, %	Main Products	Ref.
		Solvent	T (°C)	H_2_ (bar)			
5%Cu/AC	Organosolv poplar	MeOH	200	20	8.1	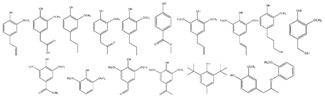	[199]
5%Ni/AC					27.9		
5%Ni- 5%Cu/AC					40.2		
5%Ni/AC	Biorefinery corncob	MeOH	240	30	12.1	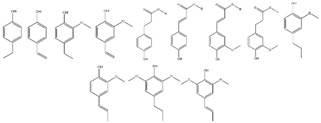	[197]
		EtOH			8.4		
5%Ru/AC	Enzymatic mild acidolysis	MeOH	240	30	39.0	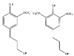	[200]
5%Pt/AC	Alkali	EtOH: H_2_O (65%)	225	40	27.6	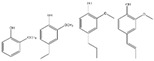	[191]
10%Ni/AC	Organosolv poplar	EtOH: IPA (1:1)	270	-	58.0	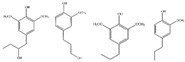	[198]
10%Ni-5%Cu/AC					63.4		
10%Ni/AC	Organosolv	MeOH	200	20	12.54	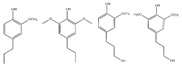	[196]
10%Fe/AC					6.3		
5%Ni-5%Fe/AC					20.3		
10%Pd/C	Enzymatic mild acidolysis	MeOH	240	30	34.0	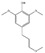	[200]

### 4.2. Graphene, N-doped and Graphene Nitride Type Carbons (g-C_3_N_4_)

**Figure 8:**
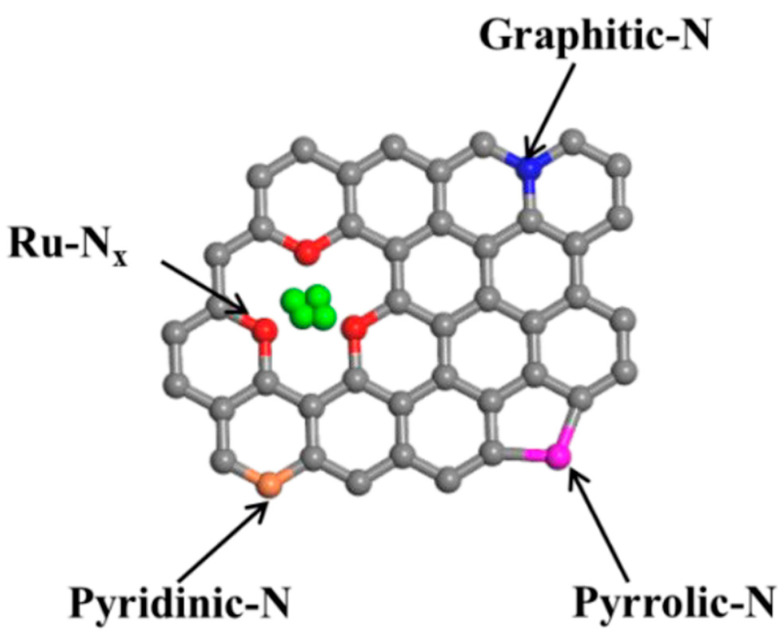
The possible bonding configurations of N atoms. Reprinted with permission from Ref. [202]. Copyright 2019, American Chemical Society.

Recently, the incorporation of heteroatom (e.g., nitrogen) in carbon-based materials has been developed, aiming to better electronic interactions between metals and supports, and thus affecting the physicochemical properties and activity of the catalysts [201]. Metal loading can be carried out either in situ, during the N-carbon synthesis, or in a following step via impregnation [201]. Ru-based catalysts supported on N-doped carbon support have been synthesized by two-stage pyrolysis at 600 and 800 °C and their activity was investigated in the hydrogenolysis of birch organosolv lignin. Under the optimized synthesis of catalysts and despite the low Ru loading (1.5 wt.%), Ru nanoparticles supported on N-doped carbon (Figure 8) resulted in 30.5 wt.% monomers. The superior activity was attributed to the high surface area, the highly dispersed and small Ru nanoparticles (2.1 nm) and the defects rich mesoporous structure, which facilitate the mass transfer and the diffusion of reactants [202]. Correlation between the catalytic activity and the synthetic parameters of the catalysts has been also observed. More specifically, the increase of pyrolysis temperature led to bigger nanoparticles, destroy of well-wrinkled morphology and a lower monomers yield. Furthermore, the absence of melamine during the synthesis of catalysts resulted to lower monomers yield (16.3 wt.%) due to limited mesoporous characteristics. It should be highlighted that the low monomers yield, which was obtained with 1.5% Ru-N-doped carbons, is still higher than the yield obtained in the presence of commercial 5%Ru/AC and 5%Pd/AC (15.7% and 14.8%) [202].

In a similar work by the same research group, birch lignin depolymerization was studied over layered graphitic carbon nitride (g-C_3_N_4_) loaded with 1.5% Ru and the highest monomer yield observed was equal to 40.7 wt.%. The high pyridinic nitrogen content, which was achieved by the addition of high melamine amount during the synthesis of the catalysts, served as metal coordination sites, leading to the stabilization of the dispersed Ru nanoparticles. This resulted in the improved metallic phase of Ru nanoparticles and the electronic interactions between N- Ru. The high catalytic activity and stability of the synthesized materials were retained after four catalytic runs, while the yield of aromatic monomers was still above 40.0% [203]. Apart from isolated lignin depolymerization, 3% Ru supported on graphite-like carbon with acidic surface formed during oxidative treatment, was also used for one-step aspen wood hydrogenolysis processes. The presence of the catalyst resulted to 49.9 wt.% monomeric phenolic compounds, accelerating the cleavage of β-O-4 and C-C linkages and C=C bonds hydrogenation [204].

### 4.3. Biomass Derived Carbons (Biochar, Lignin, etc.)

Currently, one of the key challenges in catalysis research is the development of low cost and metal-free catalysts. Alternative sources of carbon supports have been developed, such as biochar from lignocellulosic biomass. Lignin itself can serve as feedstock for the synthesis of carbon supports.

**Figure 9:**
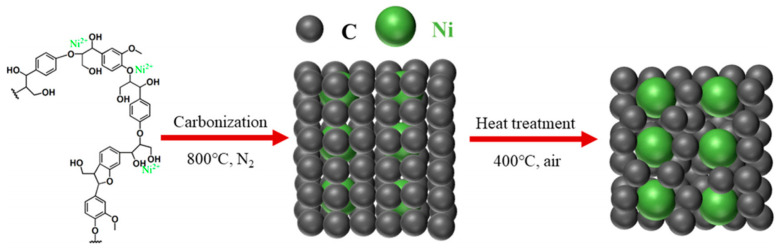
Schematic illustration of the construction of “inlaid type” catalyst. Reprinted with permission from Ref. [206]. Copyright 2019, Elsevier.

Recently, Totong et al. synthesized a solvothermal carbon as well as the sulfonated and the nitrogen-doped analogues from organosolv lignin. The catalysts were tested in the alkaline lignin depolymerization, as metal-free catalysts, and resulted in a 48–55% conversion towards alkoxy phenols compounds, with total monomers yield 7.6% [205]. Compared to the graphene oxide catalysts, lignin derived carbons exhibited higher catalytic activity while the nitrogen doped solvothermal carbon exhibited the highest conversion (55%) and yield of phenolic compounds (7.6%). Similarly, lignin was used as a carbon precursor for the synthesis of “inlaid type” Ni-based catalyst via carbonization at 800 °C and further re-expose in air, at 400 °C (Figure 9). The material prepared without re-exposure in air resulted in 70% poplar organosolv lignin conversion with 7.2 wt.% monomer yield. Increase of both conversion (87.4%) and monomer yields (23.3 wt.%) was achieved with the use of the re-exposed in air material, due to the electron effect and the large surface area. Both “inlaid” materials exhibited higher catalytic activity than the traditional hydrogenolysis catalysts Ni/C and Pd/C, with 70% and 84.4% conversion and 11.7 wt.% and 18.9 wt.%, respectively [206].

Except for lignin, lignocellulosic biomass can also serve as biochar feedstock. Biochar supported Ni–Mo_2_C mesoporous catalysts have been synthesized via pyrolysis of fir sawdust impregnated with metal precursor [207]. The bimetallic catalyst resulted in 61.3 wt.% liquid products from the hydrogenolysis of hardwood lignin, with 31.94 wt.% monomers yield (phenols, guaiacols, and trimethoxybenzenes). The catalytic performance of Ni–Mo_2_C/C is superior compared to the monometallic catalysts, Ni/C and Mo_2_C/C, which produced 47.9 wt.% and 52.1 wt.% liquid products and 19.47 wt.% and 14.56 wt.% monomers yield, respectively. Moreover, the bimetallic catalyst demonstrated better catalytic activity than the material prepared by the conventional method, which resulted in 45.7 wt.% liquid products with 12.66 wt.% monomers. The higher activity was attributed to the synergistic effect of the graphitized biochar matrix and the Ni–Mo_2_C nanoparticles, which facilitates electron transfer. Combining the ferromagnetic properties of Ni–Mo_2_C/C, the facile recovery of the catalyst from the reaction mixture and its stability, allows the reuse of the catalyst for at least five successive runs [207]. In a similar research, rice straw was efficiently converted to bio-char through slow pyrolysis at 450 °C and the obtained bio-char, after activation, was impregnated with metal precursor towards 10% Ni/AC, 10% Co/AC and 5% Ni-5% Co/AC catalysts [208]. The bimetallic catalyst led to high bio-oil yield (72 wt.%) in the liquefaction of alkali lignin in ethanol, toward vanillin production (34.8%). The enhanced catalytic activity of the bimetallic catalyst was attributed to the medium surface area and the higher acidity, compared to the monometallic Ni/AC and Co/AC.

Lignocellulosic derived monomers might be also utilized in biochar synthesis. Lama et al. reported the synthesis of two heterogeneous Ni-based catalysts supported on hierarchically porous carbon, derived via salt-melt synthesis from glucose and glucosamine (N-doped carbon). In the hydrogenolysis of Kraft lignin in batch reactors the hierarchical porosity of both catalysts enhanced the degradation of lignin towards aromatic compounds, compared to the commercial Ni/AC. Furthermore, N-doping had a beneficial effect on Ni active sites. In flow systems, the Ni-hierarchical carbons provide the possibility for more than 50 h operation, without any significant loss of activity [209].

### 4.4. MOF-Derived Carbons

Metal−organic frameworks (MOFs) have been widely used as catalyst precursors in liquid phase biomass valorization reactions, due to their wide functionality, and chemical and thermal stability, compared to the conventional supports [210]. MOF-derived carbon materials activity was examined in the hydrogenolysis of lignin-derived aryl ethers. Over Ni/N-C catalyst, synthesized by a Ni-MOF precursor at different pyrolysis temperatures, C-O bonds of aryl ethers were cleaved towards benzene and cyclohexane with 70–100% conversion [211]. Catalysts pyrolysis temperature was proved to have a crucial effect on the hydrogenolysis of the diphenyl ether, as an increase in the pyrolysis temperature from 350 °C to 450 °C led to an increase of conversion from 80.1% to 100%. However, further increase of the pyrolysis temperature to 550 °C led to lower conversions (73.0%). This trend was attributed to the smallest particles of Ni (450 °C vs. 550 °C) and the presence of active lattice planes (200) and (220) (350 °C vs. 450 °C)

**Figure 10:**
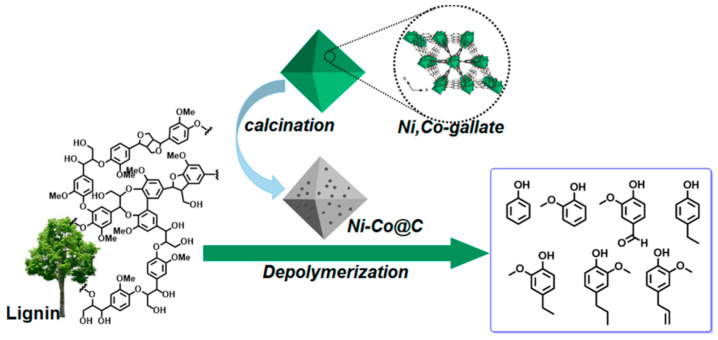
MOFs derived carbon catalysts for lignin conversion. Reprinted with permission from Ref. [212]. Copyright 2019, American Chemical Society.

Zhu et al. investigated the hydrogenolysis of poplar organosolv lignin in the presence of monometallic (Ni/C) and bimetallic (Ni-Co/C) microporous catalysts derived from gallate-based MOFs (Figure 10). The highest catalytic activity was exhibited by the bimetallic Ni_0.5_Co_0.5_/C with 55.2% monophenols yield, significantly higher than the commercial Pd and Ru/AC catalysts. Decrease of cobalt content and lower calcination temperature led to the decrease of monophenols yield [212]. One-dimensional nitrogen-doped Mo_2_C catalyst, mediated from Mo-MOF, was used in the enzymatic hydrolysis lignin depolymerization. Even in the absence of external hydrogen source, the material exhibited total monomers yield 10.1 wt.% with high selectivity toward monophenols due to Mo_2_C phase and Mo^5+^ state [213].

### 4.5. Carbides

Catalyst stability and activity are affected mainly by coke depositions. Coke is formed during the hydrogenolysis due to the repolymerization of lignin intermediates. Carbides of transition metals are considered to be quite resistant in coke formation. Molybdenum-based carbides have been extensively used in lignin hydrogenolysis. α-MoC_1-x_/AC catalyst was proved to be more active in the ethanolysis of Kraft lignin compared to the metallic molybdenum and molybdenum oxide supported on alumina [214]. Crystal phase of molybdenum carbide can significantly influence the catalytic activity. Different Mo_2_C crystal phases can be obtained under different carburization atmospheres and temperatures [215]. Under CH_4_/H_2_, a gradual increase of process temperature resulted in the gradual transformation of MoO_2_ phase to α-MoC_1−x_. The phase transformation positively affected the transfer hydrogenolysis of Kraft lignin, while the optimum carburization temperature was proved to be 600 °C. The high aromatic compounds yield (0.417 g/g lignin) is attributed to the larger surface area and the better dispersion of α-MoC_1−x_. Mixed crystal phases of α-MoC_1−x_ and β-Mo_2_C, formed via carburization in H_2_, resulted in a higher aromatic compounds yield (0.455 g/g lignin) from Kraft lignin. The highest aromatic compounds yield (0.516 g/g lignin) was determined for the catalyst obtained via carburization under N_2_ at 700 °C, due to the better crystallization of α-MoC_1−x_ and β-Mo_2_C phases and the larger surface area [215].

Undoped (Mo_2_C) and nickel-doped (Ni-Mo_2_C) molybdenum carbides were synthesized and their activity was evaluated in reductive depolymerization of corn stover lignin in water. Bare Mo_2_C resulted in 67.7% conversion and 6.1 wt.% monomers yield. Nevertheless, Ni-Mo_2_C exhibited better catalytic activity with 86.3% conversion and 7.1 wt.% monomers due to the higher number of metallic sites [216]. Enhanced catalytic activity was achieved by the physical mixture of Ni-Mo_2_C with H-Beta zeolite, which led to 88.3% lignin conversion towards 8.8% monomers. The low monomers yield was attributed to the repolymerization tendency of lignin intermediates in water. Partial substitution of water content (50%) with ethanol, which acts both as solvent and hydrogen donor, increased dramatically the yield of monomers (37.3 wt.%).

In organosolv lignin depolymerization, the catalytic activity of Mo_2_C/AC was improved by metal doping with non-precious metals. Raw Mo_2_C/AC catalyst, which was synthesized via impregnation method followed by carburization in H_2_ and was composed mainly by graphitized carbon and β-Mo_2_C, resulted in 79.52% liquid yield with 19.91% monomers [217]. Doping with one metal (Ni, Fe, Cu) dramatically increased the monomers and the bio-oil yields from 19.91 to 28.39 wt.% and 79.52% to 84.75%, respectively, while the Ni-Mo_2_C/AC exhibited the highest monomers yield. Further improvement was achieved using bimetallic catalysts. Ni-Fe-Mo_2_C/AC enhanced the monomers production (35.53 wt.%), but not the bio-oil yield (89.56 wt.%). Contrary, while Ni-Cu-Mo_2_C/AC improved the bio-oil yield (92.13%), but resulted in less monomers (25.12%). The internal interaction between Ni-Fe alloy and β-Mo_2_C/AC, combined with the large surface area (426 m^2^/g), resulted in higher lignin liquefaction and monomers production, providing a stable and reusable catalyst at least for five successive runs.

Tungsten-based catalysts were also employed for beech dioxasolv lignin depolymerization. The co-existence of tungsten trioxide and tungsten carbide crystal phases has beneficial effect on the catalytic activity, enhancing both dehydration and hydrogenolysis reactions, and resulted in 56.4 wt.% bio-oil yield with 10.8 wt.% monomers [218]. Single tungsten trioxide phase, which was formed at lower carburization temperature (500 vs. 1000 °C), exhibited lower catalytic performance with 52.3 wt.% bio-oil and 6.3 wt.% monomers yield, due to the limited metallic sites.

### 4.6. Nano-Structured Carbons (Nanotubes, etc.)

Although a wide variety of carbon nanostructures have been developed for lignin hydrogenolysis, carbon nanotubes are most commonly used. A Ru supported on multi-walled carbon nanotube catalyst was used in the reductive depolymerization of lignin-containing stillage derived from 2nd generation bioethanol production. Compared to the convectional 5%Ru/AC catalyst, 5%Ru/MWCNT increased the bio-oil yield from 60 wt.% to 68 wt.%, despite the lower surface area (300 vs. 800 m^2^/g) [219]. The improved bio-oil yield of 5%Ru/MWCNT is attributed to the higher accessibility of lignin to the metal clusters, which was limited in presence of 5%Ru/AC due to the microporous structure. However, metal particles deposited on the inner hollow structure of MWCNT are inaccessible for lignin oligomers, and consequently resulted in lower monomers yield.

The low-cost MoO_x_ supported in carbon nanotubes exhibited comparable activity to precious metal-based catalysts in the hydrogenolysis of enzymatic mild acidolysis lignin, leading to high bio-oil (63.0 wt.%) and monomer (33.0 wt.%) yields. Commercially available, 5%Ru/AC and 10% Pd/AC catalyst, resulted in higher bio-oil (73 wt.% and 76 wt.%) and similar monomer yields (39.0 wt.% and 34 wt.%). Regarding the bio-oil composition, depolymerization of lignin over MoO_x_/CNT resulted in 47.2% unsaturated substituted products, which are absent from the bio-oils produced over commercially available catalysts [200].

Catalytic activity of transition metals (Co, Ni and Fe) supported on carbon nanotubes have also been examined in the hydrogenolysis of Kraft lignin. Bare CNT support resulted in 70% conversion with 45.2 wt.% bio-oil yield and 24.8 wt.% gas yield [220]. Transition metals improved both conversion (72–82%) and bio-oil yield (56–66.2 wt.%), minimizing gas and char yields. Co/CNT catalyst exhibited the maximum conversion (82%) and bio-oil yield (66.2 wt.%), due to the highest surface area (257 m^2^/g). In contrast, Fe/CNT with lower surface area (235 m^2^/g) resulted in decreased conversion (71.1%) and bio-oil yield (56.6 wt.%). The main products were phenol, alkyl/alkoxy substituted phenols, aldehydes, ketones, carboxylic acids and aromatics. CNTs as metal-free catalysts produced mainly phenol, while transition metals enhanced the production of vanillin and alkyl substituted phenols.

A short comparison between carbon-based and other commonly used supports (zeolites, SiO_2_, Al_2_O_3_) in lignin hydrogenolysis revealed that carbon-based materials significantly enhance the lignin depolymerization (conversion, bio-oil and monomers yields). The key parameters highlighted by many researchers were the supports properties (porosity, acidity/basicity, morphology, stability, etc.) and the metal-support interactions, controlling the accessibility of lignin and the diffusion of the monomers. Sanyoto et al. attributed the higher monomers yield (27.6%) observed with 5%Pt/AC to the larger Pt surface area compared to 5%Pt/ZSM-5, which resulted to 3.4% monomers and oxidic supports with 3.8–19.5% monomers [191]. Similarly, ZrO_2_ as support for 5% Ru exhibits lower liquid bio-oil yield (61 wt.%) compared to MWCNT with 68% [219]. The effect of MWCNT support was more profound on lignin inter-unit linkages abundance. In the presence of 5%Ru/ZrO_2_, the depolymerization degree of β-O-4, β-5 and β-β linkages was 42%, 10% and 4%, respectively, while with 5%Ru/MWCNT the de-polymerization degree was 74%, 73% and 37%. Furthermore, MoO_x_/CNT catalysts exhibit higher monomers yield (33 wt.%) than the commercially available Raney Ni (28 wt.%) and significantly higher selectivity towards unsaturated compounds (47.2% vs. 2.8%) [200].

## 5. Conclusions

Carbon-based nanocatalysts (CnCs) have been extensively and successfully utilized as metal-free catalysts or as supports/additives for the synthesis of novel composites/hybrids, consisting of noble or transition metals. Depending on the targeted application, crucial physicochemical features, as for instance the morphological (size and shape) or textural properties (specific surface areas, micro and/or mesopore volume) as well as the surface chemistry (acidity, basicity, oxygen functionality), was possible to be tuned on-demand. In the case of the composite formation, the arisen synergistic effects showed to play a key role towards effective catalytic performance. This review summarizes the recent reports of the utilization of CnCs for catalytic biomass valorization and environmental remediation applications. The main carbonaceous phases are widely used commercial activated carbons, graphene/graphite and the chemical modified counterparts like graphite oxide and reduced graphite oxide, graphitic carbon nitride, carbon quantum dots, carbon nanotubes, fullerene and functionalized porous carbons (derived for instance from Metal-Organic Frameworks). CnCs revealed to possess elevated catalytic activity in the conversion/hydrogenolysis of lignin towards high bio-oil yields enriched mainly in alkyl and oxygenated phenolic monomers, production of 5-hydroxymethylfurfural (5-HMF) from cellulose or starch-rich food waste, as well as photocatalytic, sonocatalytic or sonophotocatalytic selective partial oxidation of 5-HMF to 2,5-diformylfuran (DFF). Additionally, the effectiveness CnCs for the photo-/sono-catalytically unselective decomposition of organic pollutants is presented and discussed.

Points for outlook

- The design of CnCs with enhanced metal-support interaction and high dispersion of (nano)metals and oxides targeting to the development of efficient and stable catalysts in biomass transformation processes, that usually involve liquid (aqueous or solvent) phase reactions.
- Modification of CnCs by the addition of various surface functional groups can have a positive impact on the thermo/photo/sono-catalytic efficiency.
- CnCs catalyst can be effective in order to promote the in situ generation of hydrogen from hydrogen donor molecules, aiming to limit the external gas hydrogen.
- The development of ultrasound-assisted techniques for the advanced oxidation processes (AOPs) may decrease energy consumption leading to more sustainable methods.
- The formation of composite of different semiconductor-based materials with carbon-based supports leading to a red-shift of the active phases’ optical bandgap, elevating the CnCs’ ability to utilize better visible light to boost the catalytic activity.
- The synergistic coupling of sonochemistry with heterogeneous photocatalysis is a disruptive example within the process intensification concept either for synthesizing chemicals or/and decontaminating the water.
- Utilizing CnCs for continuous flow processes has barely been studied to date, hence it will be beneficial to do so towards more cost-effective processes that could be scaled-up for real-life applications.

## Figures and Tables

**Table 1:** Sonocatalytic and sonophotocatalytic degradation of organic pollutants using characteristic CnCs.

Catalyst	Sono (S) or SonoPhoto (SP)	COMPOUND	Experimental Conditions							Degradation (%)	Ref.
			Catalytic Loading (g/L)	Frequency of Sonication (kHz)	Power of Sonication (W)	Duration (Min)	Light Source	Concentration	Temperature (°C)		
single walled carbon nanotubes (SWNTs)	S	ibuprofen	0.045	1000	180	60	-	50 mg/L	15	97	[64]
		sulfamethoxazole	0.045	1000	180	60	-	45 mg/L	15	92	
mMBiPO_4_-MWCNTs-In_2_O_3_	S	Norfloxacin	1	40	300	150	-	10 mg/L	25–28	69%	[65]
F-TiO_2_ (B)/SWCNT	SP	malachite green	0.1	45−55 Hz	285	120	500 W halogen lamp	30 mg/L	20	95	[66]
	P		0.1	-	-	120	500 W halogen lamp	30 mg/L	20	91	
TiO_2_/CNTs	SP	methyl orange	1	20	50	60	30 W black light blue lamp	25 ppm	-	66	[67]
rGO/Ag_2_CO_3_	SP	Tetracycline	0.3	20	-	60	500 W xenon light source	10 ppm	20	97	[68]
Au/BeTiO_2_/rGO	SP	Tetracycline	0.25	40	600	60	300 W halogen lamp	15	-	100	[69]
NiFe-LDH/rGO	SP	moxifloxacin	1	36	150	60	10 W LED lamp	20 mg/L	Room tem	90	[70]
ZnCr LDH/rGO	SP	Rifampicin	1.5	36	150	60	10 W LED vis	15 mg/L	Room tem	87	[71]
ZnCr LDH/BC	SP	Rifampicin	0.6	36	150	40	30 W LED vis	15 mg/L	-	98	[72]
TiO_2_/coconut shell-derived Activated carbon	S	Methylene blue	2.5	26	40	120	-	10 mg/L	25	50	[73]
TiO_2_ decorated on magnetic activated carbon (MAC@T)	SP	tetra-cycline	0.4	20	70	180	UV (254 nm)	25	20	93	[74]
TiO_2_/BC	S	Methylene blue	1.5	40	300	80	-	20	room	98	[75]
